# Epigenomic profiling of primary gastric adenocarcinoma reveals super-enhancer heterogeneity

**DOI:** 10.1038/ncomms12983

**Published:** 2016-09-28

**Authors:** Wen Fong Ooi, Manjie Xing, Chang Xu, Xiaosai Yao, Muhammad Khairul Ramlee, Mei Chee Lim, Fan Cao, Kevin Lim, Deepak Babu, Lai-Fong Poon, Joyce Lin Suling, Aditi Qamra, Astrid Irwanto, James Qu Zhengzhong, Tannistha Nandi, Ai Ping Lee-Lim, Yang Sun Chan, Su Ting Tay, Ming Hui Lee, James O. J. Davies, Wai Keong Wong, Khee Chee Soo, Weng Hoong Chan, Hock Soo Ong, Pierce Chow, Chow Yin Wong, Sun Young Rha, Jianjun Liu, Axel M. Hillmer, Jim R. Hughes, Steve Rozen, Bin Tean Teh, Melissa Jane Fullwood, Shang Li, Patrick Tan

**Affiliations:** 1 Cancer Therapeutics and Stratified Oncology, Genome Institute of Singapore, 60 Biopolis Street, Genome #02-01, Singapore 138672, Singapore; 2 Cancer and Stem Cell Biology Program, Duke-NUS Graduate Medical School, 8 College Road, Singapore 169857, Singapore; 3 NUS Graduate School for Integrative Sciences and Engineering, National University of Singapore, 5 Lower Kent Ridge Road, Singapore 119074, Singapore; 4 Cancer Science Institute of Singapore, National University of Singapore, 14 Medical Drive, #12-01, Singapore 117599, Singapore; 5 Department of Physiology, Yong Loo Lin School of Medicine, National University of Singapore, 2 Medical Drive #04-01, Singapore 117597, Singapore; 6 Department of Human Genetics, Genome Institute of Singapore, 60 Biopolis Street, Genome #02-01, Singapore 138672, Singapore; 7 Medical Research Council (MRC) Molecular Haematology Unit, Weatherall Institute of Molecular Medicine, Oxford University, Oxford OX3 9DS, UK; 8 Department of Upper Gastrointestinal & Bariatric Surgery, Singapore General Hospital, Singapore 169608, Singapore; 9 Division of Surgical Oncology, National Cancer Centre Singapore, 11 Hospital Drive, Singapore 169610, Singapore; 10 Department of General Surgery, Singapore General Hospital, Singapore 169608, Singapore; 11 Department of Medical Oncology, Yonsei University College of Medicine, Seoul 120-752, South Korea; 12 SingHealth/Duke-NUS Institute of Precision Medicine, National Heart Centre Singapore, Singapore 168752, Singapore; 13 Laboratory of Cancer Epigenome, Department of Medical Sciences, National Cancer Centre, 11 Hospital Drive, Singapore 169610, Singapore; 14 School of Biological Sciences, Nanyang Technological University, Singapore 637551, Singapore; 15 Cellular and Molecular Research, National Cancer Centre, 11 Hospital Drive, Singapore 169610, Singapore

## Abstract

Regulatory enhancer elements in solid tumours remain poorly characterized. Here we apply micro-scale chromatin profiling to survey the distal enhancer landscape of primary gastric adenocarcinoma (GC), a leading cause of global cancer mortality. Integrating 110 epigenomic profiles from primary GCs, normal gastric tissues and cell lines, we highlight 36,973 predicted enhancers and 3,759 predicted super-enhancers respectively. Cell-line-defined super-enhancers can be subclassified by their somatic alteration status into somatic gain, loss and unaltered categories, each displaying distinct epigenetic, transcriptional and pathway enrichments. Somatic gain super-enhancers are associated with complex chromatin interaction profiles, expression patterns correlated with patient outcome and dense co-occupancy of the transcription factors CDX2 and HNF4α. Somatic super-enhancers are also enriched in genetic risk SNPs associated with cancer predisposition. Our results reveal a genome-wide reprogramming of the GC enhancer and super-enhancer landscape during tumorigenesis, contributing to dysregulated local and regional cancer gene expression.

## Abstract (web summary)

Gene expression is regulated by enhancers and super-enhancers, which can be identified by chromatin profiling. Here, the authors surveyed gastric cancer samples and cell lines to identify enhancer elements exhibiting somatic alterations.

## Graphical abstract

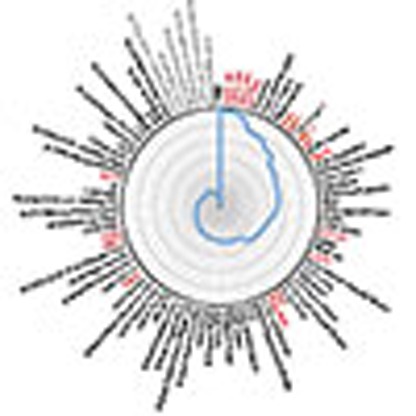

Aberrant gene expression patterns are a universal hallmark of human malignancy driving clinically important traits such as proliferation, invasion and metastasis^1^. Cancer transcriptomes can be reprogrammed by genomic alterations (somatic mutations, copy number alterations and structural variations) affecting signalling molecules and transcription factors (TFs)^2^. Besides protein-coding genes, *cis*-regulatory elements in noncoding genomic regions can also influence transcriptional programs by facilitating or restricting TF accessibility^3^. Distinct regulatory element classes are defined by different epigenomic marks, involving DNA methylation, histone methylation/acetylation and other combinations^4^. For example, promoters are marked by histone H3 lysine 4 tri-methylation (H3K4me3), enhancers by H3K4me1, active regulatory elements by H3K27ac and repressive features by H3K27me3^5^,^6^. A growing number of studies are now highlighting the significance of histone modifications in the development and maintenance of disease states^4^,^6^,^7^. It has also been proposed that because epigenetic modifications are more stable than RNA transcripts, histone modification profiling may offer a better measure of transcriptional status than RNA sequencing^8^.

Enhancers are regulatory elements localized distal to promoters and transcription start sites (TSSs). Occupying 10–15% of the human genome^9^, enhancers play important roles in cell identity and tissue-specific expression by regulating one or more genes at large distances (>1 Mb)^10^. Recent studies have demonstrated that enhancers can be subdivided into different classes, with one such class comprising enhancer clusters occurring in close proximity, referred to as ‘super-enhancers'^11^,^12^. Compared to typical enhancers, super-enhancers are larger in size, exhibit higher TF binding densities and are strongly associated with key cell identity regulators, similar to locus control regions^13^, DNA methylation valleys^14^, transcription initiation platforms^15^ and stretch enhancers^16^. Super-enhancers are also enriched in disease-associated genetic variants^11^, are acquired by cancer cells at key oncogenes^11^, and importantly are more sensitive to therapeutic perturbation^12^.

The emerging importance of super-enhancers in human disease, coupled with their exquisite tissue-specificity, raises a need for comprehensive super-enhancer catalogues of different cell types and disease conditions. In cancer, such super-enhancer catalogues are indeed emerging, for both haematological and solid malignancies^11^,^17^. However, most of these studies to date have relied on *in vitro* cultured cancer cell lines, which have two limitations. First, *in vitro* cell lines are known to experience substantial epigenomic alterations after repeated passaging^18^. Second, for many cancer cell lines, matched normal counterparts are frequently not available, complicating the ability to identify true tumor-specific somatic alterations.

Gastric cancer (GC) is the fifth most common cancer worldwide and the third leading cause of global cancer mortality^19^. Most GCs are adenocarcinomas, and molecular studies have revealed key genetic alterations associated with gastric malignancy, including mutations in chromatin modifier genes such as *ARID1A*, and amplifications in *HER2*, *FGFR2* and *MET*^20^. In contrast to genetic alterations, our knowledge of the GC epigenome remains poorly understood, with current knowledge largely confined to patterns of aberrant DNA methylation^21^.

To address the role of histone modifications in GC, we recently reported a proof-of-concept study employing micro-scale chromatin profiling (Nano-ChIPseq) to survey histone modifications in primary GCs^4^. In this study, we extended this effort to characterize the GC super-enhancer landscape. By integrating predicted super-enhancer data from both cell lines and primary tumours, we provide evidence that like enhancers, predicted super-enhancers are heterogeneous and can be further subclassified into biologically distinct subgroups with impact on tumour-specific gene expression, cancer hallmarks and disease genetic variation. We also found that in GCs, predicted super-enhancers exhibiting somatic gain are associated with CDX2 and HNF4α occupancy. Taken collectively, our results reveal a pervasive reprogramming of the gastric super-enhancer landscape during tumourigenesis, underpinning wide-spread alterations in cancer gene expression.

## Results

### Distal predicted enhancer landscapes of GC cell lines

Using Nano-ChIPseq, we generated 110 chromatin profiles from 19 primary GCs, 19 matched normal gastric tissues and 11 GC cell lines covering multiple histone H3 modifications (H3K27ac, H3K4me3, H3K4me1) (average ∼3.3 × 10^7^ reads per profile). Clinical information and molecular classification of the primary GCs is presented in Supplementary Table 1, sequencing statistics in Supplementary Data 1 and clinico-pathological details for the GC lines in Supplementary Table 2. Our series included 10 gland forming adenocarcinomas (53%, intestinal type), 6 samples with highly infiltrating isolated cells (32%, diffuse type) and 3 GC samples (15%) of mixed histology. More than 60% of the tumours (*n*=12) were Stage 3 or above (AJCC 7th edition). We performed extensive quality control analysis of the Nano-ChIPseq data, including variations in mapping quality filters, analysis of biological replicates and promoter ChIP-enrichment, and assessment by the quality control software CHANCE (CHip-seq ANalytics and Confidence Estimation^22^). Increasing mapping threshold stringencies (from MAPQ ≥10 to 20) did not appreciably alter mapping statistics: >90% of the total mapped reads were retained, and 85% of ChIP-enriched peaks and 98% of predicted enhancers were rediscovered respectively (Supplementary Fig. 1). Histone peak concordance between biological replicates of KATO-III cells generated by Nano-ChIPseq, and also against independent KATO-III H3K27ac data generated by conventional ChIP-seq^23^, confirmed high reproducibility (overlaps of ∼85 and ∼90%) (Supplementary Fig. 2). Comparisons of input and input-corrected H3K27ac and H3K4me3 signals at 1000 promoters associated with highly expressed protein-coding genes revealed successful enrichment in 48 out of 50 (96%) H3K27ac and 42 out of 42 (100%) H3K4me3 libraries respectively. CHANCE analysis of ChIP enrichment, particularly for H3K4me1 (which is depleted at promoters), revealed that the large majority (85%) of samples exhibited successful enrichment (Methods). These results demonstrate the good technical quality of the Nano-ChIPseq cohort. Besides Nano-ChIPseq, the samples were also processed for DNA methylation analysis (Infinium HumanMethylation 450 K BeadChip arrays), copy number analysis (Affymetrix SNP arrays) and Illumina RNA-sequencing.

**Figure 1:**
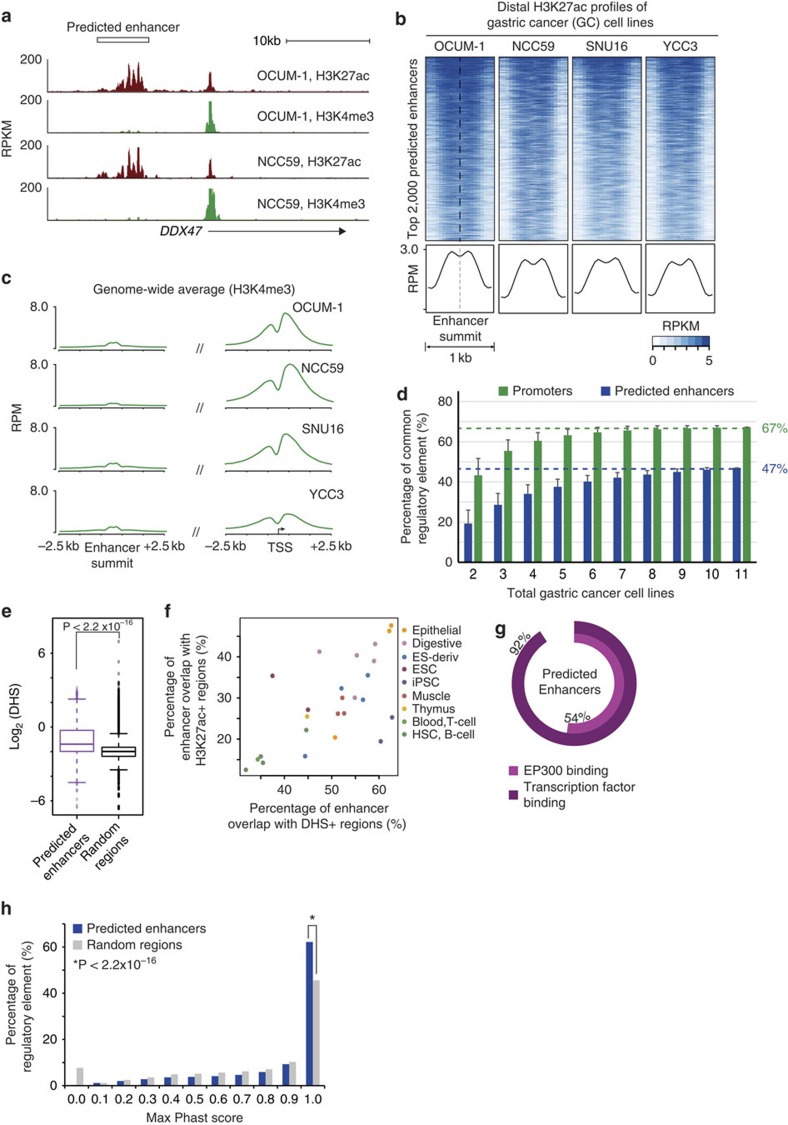
Distal Predicted Enhancer landscapes of GC cell lines. (**a**) Histone profiles of OCUM-1 and NCC59 GC cells show enrichment of H3K27ac and H3K4me3 around the *DDX47* TSS. A predicted enhancer element exhibiting H3K27ac enrichment and >2.5 kb away from the *DDX47* TSS was identified. (**b**) Snapshot of distal H3K27ac profiles in 4 of the 11 GC cell lines, visualizing the activity of the top 2,000 predicted enhancers and the genome-wide average H3K27ac signal around the predicted enhancers. (**c**) Genome-wide average H3K4me3 signals around predicted enhancers and active TSSs in GC cell lines. (**d**) Recurrence rates of regulatory elements. Data presented are the mean percentage +/− standard deviation of common regulatory elements (enhancer—blue; promoter—green) found in two or more gastric cancer cell lines, as a function of number of cell lines. (**e**) Chromatin accessibility of predicted enhancers versus randomly selected regions. DNase I hypersensitivity (DHS) data from normal gastric tissues^28^ was used as a surrogate. The distribution of DHS signals was tested using a one-side Welch's *t*-test for statistical significance. (**f**) Percentage of overlap between predicted enhancers, chromatin accessible regions (denoted as DHS+, *x* axis) and active regulatory elements (denoted as H3K27ac+, *y* axis) from 50 epigenomic profiles originating from nine different tissue/cell categories. (**g**) Percentage of predicted enhancers overlapping with EP300 and transcription factor binding sites. (**h**) Distribution of maximum Phast scores (a measure of DNA sequence conservation) in predicted enhancers and randomly selected regions.

We chose to use GC cell lines as a discovery cohort to discover cancer-associated distal enhancers in GC, as cell lines are purely epithelial in nature, have the highest data quality, and because previous studies have shown that stromal contamination in primary tissues can influence genomic results^24^. We also focused on recurrent epigenetic alterations present in multiple GC samples, which reduces the introduction of ‘private' epigenetic alterations associated with individualized cell line features. First, we mapped genome-wide *cis*-regulatory elements based on H3K27ac signals, previously shown to mark active promoters and enhancers^4^. To enrich for enhancer elements, we focused on H3K27ac signals located distant from known annotated TSSs (TSSs; >2.5 kb) in accordance with previous studies^25^ (Fig. 1a). We then further refined the enhancer predictions using aggregated H3K4me1 and H3K4me3 data, excluding from analysis predicted enhancers exhibiting high H3K4me3/H3K4me1 log ratios (>2.4)^26^. Using this approach, we identified 3,017 to 14,338 putative distal enhancers in the GC lines (Fig. 1b), with an average genomic footprint of 25 Mb/line.

In total, we detected 36,973 predicted distal enhancer regions, spanning ∼140 Mb or approximately 5% of the human genome. The predicted enhancers exhibited a bimodal H3K27ac signal distribution^11^ (Fig. 1b), were depleted of H3K4me3 and were enriched in H3K4me1 signals, similar to enhancer modification profiles reported in previous studies^6^ (Fig. 1c and Supplementary Fig. 3). Visual comparison of selected H3K27ac-enriched regions revealed that some regions are active in multiple lines (‘recurrent') while other regions are active in only one line (‘private'). These observations are similar to previous results in both lymphoblastoid^27^ and colon carcinoma cell lines^7^. Approximately 47% of the predicted enhancers were recurrent, exhibiting activity in at least two GC cell lines (Fig. 1d). The percentage of recurrent enhancers was significantly lower compared to promoters (67 versus 47%, *P*<2.2 × 10^−16^, one-sided proportion test), indicating that enhancer activity is highly variable across GC cell lines.

We validated the predicted enhancers by integrating publicly available epigenomic datasets. Using DNase I hypersensitivity (DHS) data of normal gastric tissues from the Epigenome Roadmap, we found that DHS signal distributions (log-transformed RPKM) at predicted enhancers were significantly greater than randomly selected regions (*P*<2.2 × 10^−16^, one-sided Welch's *t*-test; Fig. 1e, Methods), indicating that predicted enhancers are associated with open chromatin. When compared against DHS and H3K27ac data of nine different tissue and cellular categories^28^, predicted enhancers exhibited the highest overlap with DHS-positive and H3K27ac-positive regions from digestive and epithelial tissues (fetal intestine, gastric and small intestine), and were distinct from non-epithelial tissue types such as blood and T-cells (Fig. 1f). Supporting their regulatory potential, 54% of the predicted enhancers (*n*=20,127) were associated with EP300 binding sites (Fig. 1g; *P*<0.001, empirical test), and 92% with TF binding sites. At the DNA sequence level, 63% of the predicted enhancer sequences were evolutionarily conserved (Fig. 1h; *P*<0.0001, empirical test).

### Super-enhancers are enriched in cancer signatures

**Figure 2:**
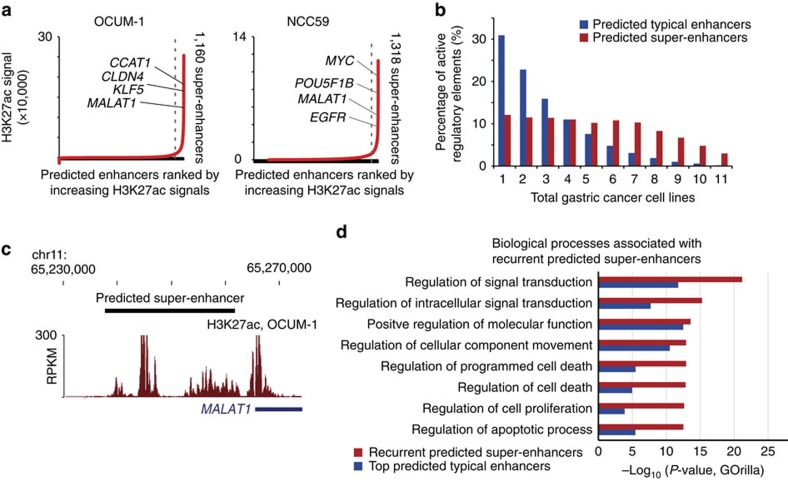
GC cell-line-derived predicted super-enhancers. (**a**) Distribution of H3K27ac ChIP-seq signals reveal locations of predicted super-enhancers showing unevenly high H3K27ac signals. Known cancer-associated genes proximal to predicted super-enhancers are indicated. Two cell lines are shown. (**b**) Percentage of distal regulatory elements (predicted typical enhancers—blue, predicted super-enhancers—red) showing H3K27ac enrichment above randomly selected regions (>99%) across increasing numbers of GC cell lines. (**c**) H3K27ac ChIP-seq signals at the *MALAT1* locus shows stretches of predicted enhancers, corresponding to a predicted super-enhancer (in filled box) with high H3K27ac signals. (**d**) Examples of top significantly associated biological processes associated with recurrent distal regulatory elements (predicted super-enhancers and top predicted typical enhancers). Negative log-transformed raw *P*-values from GOrilla were used.

‘Super-enhancers' or ‘stretch-enhancers' refer to a sub-population of enhancers displaying extended physical proximity (+/− 12.5 kb), and have been proposed to be critical for cell identity and maintenance of healthy or disease state^11^,^12^. Using the ROSE algorithm^12^, we identified 133 to 1318 predicted super-enhancers per GC line, collectively encompassing 3,759 non-redundant-predicted super-enhancers (Fig. 2a). We thus estimate that about 10% of GC cell-line-predicted enhancers are associated with predicted super-enhancer activity, consistent with the 4–19% estimated by other studies^11^. Compared to predicted typical enhancers, predicted super-enhancers exhibited a significantly greater tendency to be recurrent (Fig. 2b; one-sided proportion test, *P*<2 × 10^−16^), with 3,345 predicted super-enhancers being active in at least two GC cell lines. We observed predicted super-enhancers associated with known protein-coding GC oncogenes (for example, *MYC* and *KLF5;* Supplementary Fig. 4a) and also at non-protein-coding gene regions such as at the *MALAT1* locus (Fig. 2c), which encodes a long-noncoding RNA (lncRNA) recently shown to promote GC proliferation^29^.

We assigned predicted super-enhancers to target genes based on regions exhibiting the nearest active TSS (defined as H3K27ac enrichment at promoters, within 500 bp of an annotated TSS). This strategy has been previously used in other studies^11^. Consistent with other reports^26^, only 53% of our predicted super-enhancer/gene interactions involved the closest proximal gene (see Methods, mean distance 76 kb). We validated the predicted super-enhancer/gene assignments using three orthogonal interaction data sets: (i) pre-determined interactions predicted by PreSTIGE^30^, (ii) GREAT^31^, and (iii) published RNAPII ChIA-PET data (encodeproject.org, GSE72816). Of 2,677 predicted interactions with protein-coding genes, 88% were supported by at least one of these three data sets (Supplementary Fig. 5). This number is likely a lower limit as the biological samples for the latter validation data in (i)-(iii) did not involve gastric tissues (see subsequent sections). To understand biological themes associated with the predicted super-enhancers, we applied GOrilla pathway analysis^32^ and found that biological processes plausibly related to cancer development, such as regulation of signal transduction, programmed cell death and cell proliferation were strongly associated with predicted super-enhancer linked genes (*P*-values 6.7 × 10^−22^ to 2.3 × 10^−13^, hypergeometric test by GOrilla) (Fig. 2d). Many of these processes (eg, regulation of programmed cell death, cell proliferation) remained significantly associated when the recurrent predicted super-enhancers were analysed by GREAT, indicating that that these enrichments are not due to biases toward genes flanked by large intergenic regions (Supplementary Fig. 6). Similar analyses employing genes linked to the top predicted typical enhancers yielded a lesser degree of enrichment (Fig. 2d). Predicted super-enhancer associated genes were also enriched for oncogenes (*P*=1.7 × 10^−8^, one-sided Fisher's exact test). When correlated to gene expression, genes associated with recurrent predicted super-enhancers and typical enhancers were both significantly correlated with RNA expression (Supplementary Fig. 4b).

### Super-enhancer heterogeneity in primary tumours

**Figure 3:**
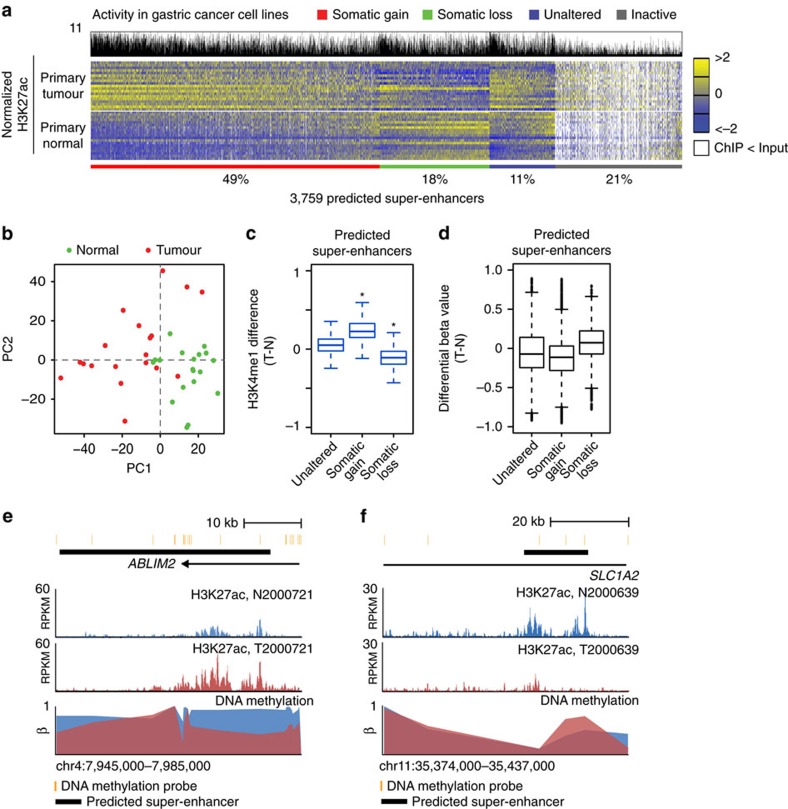
Somatic predicted super-enhancers in primary GCs and matched normal samples. (**a**) Activity of cell-line-derived predicted super-enhancers in 19 primary tumour and matched normal samples. H3K27ac predicted super-enhancer signals in units of column-transformed RPKM values (*z*-score) were visualized. The frequency of active predicted super-enhancers in GC lines *in vitro* is presented as the top histogram (black, above the heatmap). Predicted super-enhancers were categorized into somatic gain, somatic loss, unaltered and inactive. In each category, the predicted super-enhancers were ordered (left to right) by their decreasing mean difference between the tumour and the normal samples. (**b**) Principal component analysis using recurrent somatic gain predicted super-enhancer signals establish a separation between tumour and normal samples. (**c**) Differences in H3K4me1 (T-N) signals (RPKM) using H3K4me1 profiles from five tumour and matched normal samples in three predicted super-enhancer categories: somatic gain, somatic loss and unaltered. *P<2.2 × 10^−16^, one-sided Welch *t*-test. (**d**) Differential *β* values in predicted super-enhancers indicate the state of methylation: hypermethylation (>0) or hypomethylation (<0) between tumours and matched normal samples. (**e**) DNA hypomethylation in a somatic gain predicted super-enhancer at the *ABLIM2* locus. (**f**) DNA hypermethylation in a somatic loss predicted super-enhancer at the *SLC1A2* locus.

To determine which cell-line-predicted super-enhancers are also associated with somatic alterations *in vivo*, we compared H3K27ac enrichment levels for these regions across 19 primary GCs and matched normal gastric tissues. While previous studies have suggested the presence of distinct molecular subtypes of GC^33^,^34^,^35^, due to limited sample sizes we elected in the current study to focus on predicted enhancer differences conserved in multiple GC tissues relative to matched normal tissues (see Discussion). Prior to analysis, we confirmed that the primary gastric normal samples were indeed reflective of gastric epithelia, by correlating against published profiles (Supplementary Discussion). Of 3,759 cell-line-predicted super-enhancers, two-thirds exhibited differential enrichment between tumours and matched normal samples (Fig. 3a, Supplementary Data 2, referred to as somatically altered thereafter). Close to half of the predicted super-enhancers (*n*=1748; 47%) exhibited somatic gain in two or more primary GCs (> 2-fold enrichment in tumour, minimum 0.5 RPKM difference), and principal component analysis (PCA) using these gained predicted super-enhancers confirmed separations between GC and matched normal tissues (Fig. 3b). Supporting the consistency of these results, the vast majority of these recurrent somatic gain predicted super-enhancers (85%, >1.5-fold change threshold) were rediscovered when using only those normal/tumour (N/T) primary pairs passing all quality control criteria (14 pairs, see earlier). Unexpectedly, despite their activity in cancer cell lines, a substantial proportion of predicted super-enhancers (18%) were associated with somatic loss rather than gain in primary GCs (Fig. 3a). It is possible that these latter regions may represent regions epigenetically silenced in primary tumours but reactivated in cell lines during *in vitro* culture (Supplementary Fig. 7a). In all, 11% of the predicted super-enhancers (*n*=416) exhibited unaltered H3K27ac levels between GCs and normal tissues (Fig. 3a, Supplementary Fig. 7b), consistent with these regions not being cancer-associated but related to ‘housekeeping' or general tissue functions. Finally, 21% (*n*=808) of cell-line-predicted super-enhancers did not exhibit sufficient H3K27ac enrichment (RPKM<0.5) in primary samples for analysis (Supplementary Fig. 7c). Interestingly, this class was also associated with low recurrence in GC lines (Fig. 3a—histogram in black). Taken collectively, these results demonstrate that predicted super-enhancers derived from cell lines can be further subclassified using histone modification data from primary tumours and matched normal controls into at least three categories—somatic gain, somatic loss and unaltered. A list of the top 100 somatic predicted super-enhancers is presented in Supplementary Data 2.

Supporting their biological distinctiveness, predicted super-enhancers belonging to the three categories also exhibited other epigenetic differences *in vivo*. For example, predicted super-enhancer alterations in H3K27ac were similarly correlated with H3K4me1 enhancer mark alterations (Fig. 3c), and at the DNA methylation level somatic gain predicted super-enhancers exhibited significantly lower DNA methylation levels, while somatic loss super-enhancers exhibited increased DNA methylation (*P*=3.8 × 10^−229^, one-sided Welch's *t*-test). Unaltered predicted super-enhancers occupied an intermediate range (Fig. 3d). As a visual example, we observed decreased DNA methylation (indicated by a lower beta value) in GC T2000721 compared to its matched normal (N2000721), mapping to a somatic gain predicted super-enhancer at the *ABLIM2* locus (Fig. 3e). In contrast, somatic loss of H3K27ac signals at a *SLC1A2* predicted super-enhancer in T2000639 showed increased DNA methylation compared to N2000639 (Fig. 3f). These results further support the biological and molecular heterogeneity of predicted super-enhancers in gastric tissues.

### Super-enhancers exhibit complex chromatin interactions

**Figure 4:**
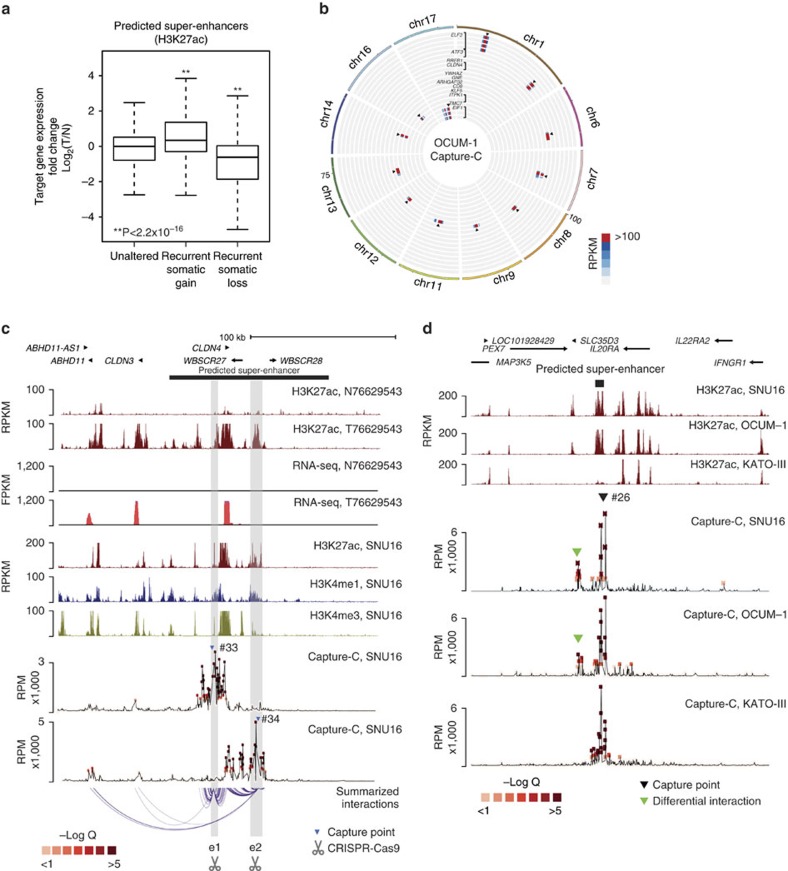
Associations between somatic predicted super-enhancers with gene expression and chromatin interactions. (**a**) Correlation between log-transformed fold changes in gene expression between different classes of predicted super-enhancers (unaltered, somatic gain, somatic loss) and predicted target gene expression. (**b**) Interaction heat map from 20 capture points covering 12 somatic gain predicted super-enhancers. Each ring represents a profile from a single capture point, denoted by a black arrowhead. Locations of the predicted super-enhancers are indicated by the gene loci in each ring. Genome-wide interaction signals were computed across the genome in 100 kb bins. Signals at regions within 2 million bases flanking the capture points were visualized. (**c**) Example of a somatic gain predicted super-enhancer at the *CLDN4* locus and interactions with neighbouring genes. Somatic gain activity is associated with upregulation of *CLDN4* and neighbouring genes (*CLDN3* and *ABHD11*) in primary GCs. Interactions were detected in SNU16 cells using two capture points, #33 and #34 by Capture-C. Summarized interactions (*Q*<0.05, r3Cseq) are presented as the last track. Two constituent predicted enhancers, e1 and e2, were deleted independently in SNU16 cells using CRISPR/Cas9 genome editing. (**d**) Correlation between predicted super-enhancer activity and long-range interactions. Long-range interactions (green triangle) to the *SLC35D3* promoter were detected with a predicted super-enhancer active in SNU16 and OCUM-1 cells. Such interactions were not observed in KATO-III cells where the predicted super-enhancer was also not detected.

Integration with copy number data revealed that the majority of somatic predicted super-enhancers are localized to copy number neutral regions (Supplementary Fig. 8a-c, Supplementary Discussion). To examine associations between predicted super-enhancers and gene expression, we interrogated RNA-seq information from the same primary samples, using the same predicted super-enhancer/gene assignments as the previous pathway analyses (Fig. 2). Somatic gain predicted super-enhancers were associated with elevated gene expression relative to matched normal samples, while somatic loss predicted super-enhancers were associated with decreased expression (*P*<2.2 × 10^−16^, one-sided Welch *t*-test; Fig. 4a).

Previous research has also shown that enhancers are often involved in long-range chromatin interactions that may influence the expression of multiple genes^10^,^26^. To identify long-range interactions associated with somatic predicted super-enhancers in GC, we applied Capture-C technology^36^ to survey interactions for 36 predicted super-enhancers, selected from regions exhibiting both recurrent somatic gains in primary tumour samples and also demonstrating activity in GC lines. Analysing three GC cell lines (OCUM-1, SNU16, KATO-III), we probed multiple genomic locations (*n*=92, referred to as ‘capture points') across the 36 predicted super-enhancers, identifying 88 capture points with significant interactions (*Q*<0.05, r3Cseq package^37^). Fig. 4b depicts 12 representative predicted super-enhancers covering 20 capture points. On average, each predicted super-enhancer exhibited 20–26 and 5–7 interactions with other genomic locations and promoters respectively. The average distance between capture points and detected interactions was approximately 17.0 kb (standard deviation: 30.5 kb). We also identified longer-range interactions, including a predicted super-enhancer interaction with the *TM4SF4* promoter at a distance of ∼100 kb in OCUM-1 cells (Supplementary Fig. 9). Notably, for regions with informative interaction data, the availability of experimental Capture-C information also allowed us to further validate 93% (*n*=62) of our original predicted super-enhancer/gene interactions. Integration of expression data from the cell lines revealed that ∼70% of the interacting promoters are associated with detectable gene expression (FPKM>0).

As a representative example, Fig. 4c depicts the long-range interaction landscape of the *CLDN4* genomic region in SNU16 cells (see Supplementary Fig. 10 for other examples). This region was selected as *CLDN4* expression has been previously associated with GC progression and prognosis^38^, and recurrent gain of the *CLDN4* predicted super-enhancer was observed in multiple primary GCs (Supplementary Fig. 8d). Specifically, we sought to investigate interactions involving two predicted sub-super-enhancer regions exhibiting high H3K27ac signals and also CDX2 and HNF4α co-binding (see later). Besides interactions with the *CLDN4* promoter, interactions were also detected with other distal promoters (up to ∼100 kb) such as *WBSCR27*, *CLDN3, ABHD11* and *ABHD11-AS1*. *ABHD11-AS1* is a long non-coding RNA was previously shown to be highly expressed in gastric cancer^39^. To validate the Capture-C data, we also performed circularized chromosome conformation capture assays (4C)^40^ on four selected predicted super-enhancers in two GC lines (OCUM-1, SNU16) (Supplementary Fig. 11). We observed a concordance between Capture-C and 4C data of 75%, similar to concordance rates between 4C experimental replicates^37^ (Supplementary Fig. 12). Due to the significantly greater depth of 4C sequencing, we also identified additional interactions, such as a long-range interaction between a predicted super-enhancer and the *KLF5* promoter at a distance of ∼350 kb (Supplementary Fig. 11b).

Previous reports have suggested that certain long-range interactions are associated with super-enhancer activity, while other interactions are more invariant and reflective of cell lineage^10^,^41^. In agreement with these findings, of 22 (out of 36) predicted super-enhancers displaying differential activity between the GC lines, four predicted super-enhancers exhibited a good correlation between predicted super-enhancer activity and the presence of long-range interactions (Fig. 4d and Supplementary Fig. 13). For the remaining 18 predicted super-enhancers, long-range interactions were observed independent of predicted super-enhancer activity.

To investigate a causal role between the predicted super-enhancers and gene expression, we used CRISPR/Cas9 genome editing to delete two enhancer regions (e1 and e2; see Fig. 4c) within the *CLDN4* predicted super-enhancer region. After confirming CRISPR deletion efficiencies in OCUM-1 and SNU16 cells (Supplementary Fig. 14a–c), we compared predicted target gene expression levels between enhancer-deleted and wild-type cells by RT-qPCR. In both cell lines, e1 CRISPR-deletion caused downregulation of multiple *CLDN4* locus genes, including *ABHD11*, *CLDN3* and *CLDN4* (*CLDN4* in SNU16 cells, Supplementary Fig. 14d). In a similar fashion, we also observed *ABHD11*, *CLDN3* and *CLDN4* downregulation after e2 deletion in OCUM-1 cells (e2-deleted SNU16 cells were not viable, hence precluding gene expression analysis; Supplementary Fig. 14e). To extend these results, we then CRISPR deleted two other predicted enhancer elements (e3 and e4) from the *ELF3* predicted super-enhancer in OCUM-1 cells (Supplementary Figs 11a and 14c), as *ELF3* has been reported as a cancer gene in several malignancies^42^. Both e3 and e4 deletion resulted in downregulation of multiple *ELF3* locus genes including *ARL8A, ELF3, RNPEP* and *TIMM17A* (Supplementary Fig. 14f). Taken collectively, these results support a causal relationship between predicted super-enhancer activity and tumour gene expression.

### Somatic super-enhancers and clinical outcome

**Figure 5:**
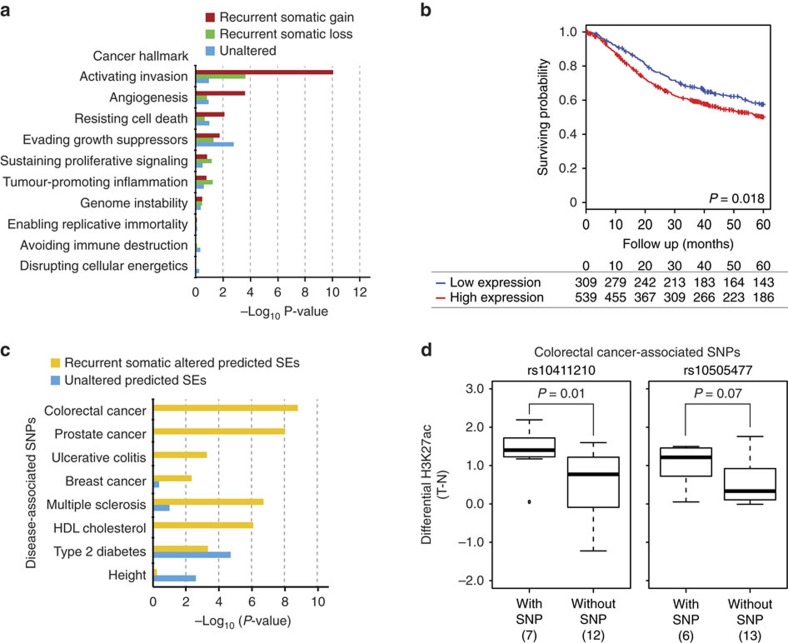
Somatic predicted super-enhancers inform patient survival and disease risk. (**a**) Cancer hallmark analysis using predicted super-enhancers showing recurrent somatic gain, recurrent somatic loss and unaltered H3K27ac signals. Negative log-transformed *P*-values from the one-sided Fisher's exact test were used. (**b**) Survival analysis comparing patient groups with samples exhibiting low (blue) and high (red) expression from genes associated with top recurrent somatic gain predicted super-enhancers. The signature is prognostic in the compilation of 848 GC patients (*P*=1.8 × 10^−2^, log-rank test), with worse prognosis observed for patients with tumours having high signature expression (hazard ratio, 95% confidence interval: 1.30 (1.05–1.61); Cox regression *P*-value after correcting for stage, age, patient locality and Lauren's histological subtypes=4.4 × 10^−2^). Survival data are indicated for every 10 months. (**c**) Enrichment of disease-associated SNPs in predicted super-enhancers. Enrichments were tested on two classes of predicted super-enhancers: recurrent somatic altered and unaltered predicted super-enhancers using chi-square test. Only diseases/traits with at least 10 SNPs found in all predicted super-enhancers were analysed. (**d**) Differential H3K27ac signals in predicted super-enhancers with and without colorectal cancer associated SNPs. The total number of patients with or without the SNPs is indicated in brackets. The difference between the two groups was tested using one-sided Welch *t*-test.

To further explore the biological and clinical relevance of predicted super-enhancer heterogeneity, we performed cancer hallmark analysis categorized by somatic modification status (gained, lost, unaltered). Of ten cancer hallmarks^11^, somatic gain predicted super-enhancers were significantly enriched in genes related to invasion (*P*=8.6 × 10^−11^, one-sided Fisher's exact test), angiogenesis (*P*=2.4 × 10^−4^, one-sided Fisher's exact test) and cell death resistance (*P*=7.8 × 10^−3^, one-sided Fisher's exact test), exceeding somatic loss and predicted unaltered super-enhancers by an order of magnitude (Fig. 5a). These results suggest that somatic gain predicted super-enhancers may be involved in traits associated with aggressive GC. When compared against predicted super-enhancer profiles of 86 cell and tissue samples, >60% of somatic gain predicted super-enhancers in GC exhibited high tissue-specificity. We also observed significant overlaps (*P*<0.001, empirical test) with predicted super-enhancers previously described in other cancer types, such as colorectal, breast, cervical and pancreatic cancer (Supplementary Fig. 15), suggesting that certain GC-associated predicted super-enhancers may also be active in other cancer types.

We next asked if gene expression patterns associated with the somatic gain predicted super-enhancers might be associated with GC patient survival. We selected genes associated with the top 50 predicted super-enhancers, from regions exhibiting both recurrent somatic gains in multiple GC patients, and also exhibiting the highest correlations with target gene expression. Supporting the validity of this approach, several genes selected in this manner were observed to have been previously shown to be overexpressed in GC, such as *CDH17*^43^ and *CCAT1*^44^. The gene list also included potentially novel GC associated genes, such as *SMURF1* and *LINC00299* (Supplementary Table 3).

We performed survival analysis across three non-Asian GC and four Asian GC cohorts comprising of 848 GC patients. Patients with GCs exhibiting high expression of predicted super-enhancer associated genes showed poor overall survival compared with GC samples where these genes are relatively lowly expressed (Fig. 5b, *P*=1.8 × 10^–2^, log rank test). Supporting the robustness of this association, the relationship with patient survival remained significant even after varying the number of predicted super-enhancers (*n*=30, *P*=0.02, log rank test; *n*=60, *P*=0.03, log rank test). In a multivariate analysis, the association with survival also remained statistically significant even after adjusting for other risk factors, such as age, stage, patient locality and histological subtype (*P*=0.044, Wald test). These data indicate that genes driven by somatic gain predicted super-enhancers in GC may be clinically important.

To address the relationship between the different predicted super-enhancer categories and disease risk, we considered previous genome-wide association studies (GWAS) studies showing that disease-associated single-nucleotide polymorphisms (SNPs) are enriched at regulatory elements^11^,^45^. We mapped catalogues of disease-associated SNPs reported from 1,470 GWAS against those predicted super-enhancers exhibiting recurrent somatic alterations (gained or lost) or unaltered predicted super-enhancers. Somatic predicted super-enhancers were enriched for disease-risk SNPs associated with various cancers (prostate, colorectal, breast; enrichment ratio=3.0–7.2; *P*<4.4 × 10^−3^, chi-square test) and gastrointestinal diseases such as ulcerative colitis (enrichment ratio=3.3; *P*=5.2 × 10^−4^, chi-square test) (Fig. 5c). In contrast, unaltered predicted super-enhancers did not exhibit similar enrichments. Unexpectedly, we also observed enrichment of multiple sclerosis SNPs in somatic altered predicted super-enhancers (enrichment ratio=4.3; *P*=1.8 × 10^−7^, chi-square test), suggestive of interconnections between cancer and autoimmune response^46^. To explore if predicted super-enhancer disease SNPs might be associated with local changes in chromatin modification, we then focused on SNPs associated with colorectal cancer reported in at least two studies and also exhibiting heterozygosity in at least 1/3 of the GC patients (see Discussion). Two SNPs fulfilled these criteria (rs10411210 and rs10505477). Samples with the rs10411210 SNP exhibited significantly higher H3K27ac signals in tumours versus matched normals (Fig. 5d; *P*=0.01, one-sided Welch's *t*-test), and a similar trend was also observed in samples with the rs10505477 SNP (*P*=0.07, one-sided Welch's *t*-test). Such associations suggest a relationship between disease-associated risk SNPs and cancer-associated histone modification.

### Super-enhancers exhibit dense TF occupancy

**Figure 6:**
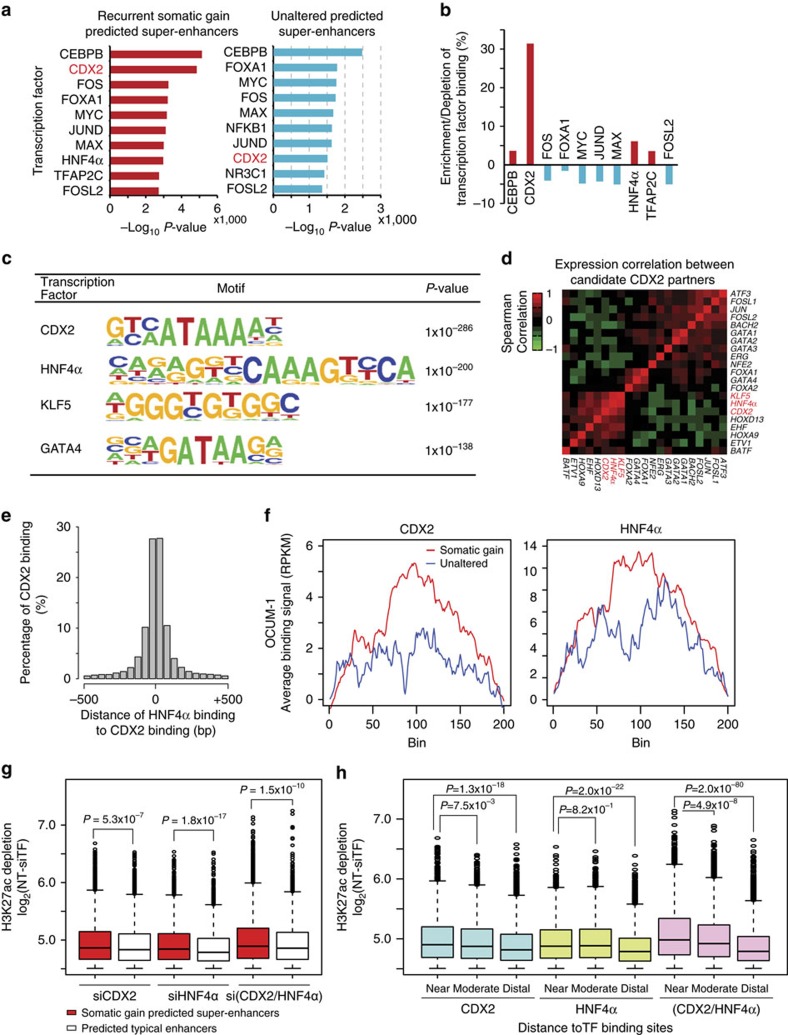
Somatic gain predicted super-enhancers in GC are associated with CDX2 and HNF4α occupancy. (**a**) Top 10 transcription factor binding enrichments at recurrent somatic gain predicted super-enhancers and unaltered predicted super-enhancers using the ReMap database. (**b**) Enrichment or depletion of ReMap transcription factors at recurrent somatic gain predicted super-enhancers compared to unaltered predicted super-enhancers. (**c**) Detection of candidate CDX2 binding partners using CDX2 binding sites and *de novo* HOMER motif identification. (**d**) Pairwise expression correlations of CDX2 and top 20 CDX2 candidate binding partners using RNA-seq from 19 primary tumour and matched normal samples. (**e**) Percentage of CDX2 binding sites co-occurring with HNF4α binding sites within a 500 bp window in OCUM-1 cells. (**f**) Differential CDX2 (left) and HNF4α (right) average binding signal analysis between recurrent somatic gain predicted super-enhancers and unaltered predicted super-enhancers. The predicted super-enhancers were also active in OCUM-1. (**g**) Distribution of H3K27ac depletion magnitude in between somatic gain predicted super-enhancers and predicted typical enhancers in OCUM-1 cells, for single and double TF silencing. Statistical significance was evaluated using the one-sided Wilcoxon rank sum test. (**h**) Association between H3K27ac sub-regional depletion in somatic gain predicted super-enhancers relative to CDX2, HNF4α or CDX2/HNF4α co-binding sites. Distances were uniformly distributed into three categories: near, moderate and distal to the binding sites. Statistical significance was evaluated using one-sided Wilcoxon rank sum test.

Finally, we explored *trans*-acting factors associated with somatic gain predicted super-enhancers. Concordant with previous studies^47^, GC predicted super-enhancers exhibited significantly enriched ENCODE TF binding profiles compared to other genomic regions, supporting the former as TF ‘hot-spots' (*P*<2.2 × 10^−16^, one-sided proportion test). Interrogating the ReMap database^48^, we then identified specific TFs associated with the different predicted super-enhancer categories. Both somatic gain and unaltered predicted super-enhancers exhibited enrichments in CEBPB, MYC and FOXA1 binding. However, among the top ten enriched TFs, CDX2 exhibited elevated enrichment in somatic gain predicted super-enhancers (rank #2), with an approximately 30% increased binding density compared to unaltered predicted super-enhancers (rank #8) (Fig. 6a,b).

As TFs often act in a cooperative manner, we then identified potential CDX2 partners by using HOMER^49^, a *de novo* motif discovery algorithm. HOMER analysis identified HNF4α, KLF5 and GATA4 binding motifs associated with CDX2 binding (Fig. 6c). We also analysed CDX2 co-binding motifs using PScanChIP^50^ with JASPAR 2016. Using PScanChIP, we predicted 367 proteins as potential CDX2 partners, once again including HNF4α, KLF5 and GATA4 (Supplementary Data 3). Gene co-expression analysis revealed that *HNF4*α (Spearman correlation, *r*=0.80) and *KLF5* (*r*=0.58) are the most strongly correlated candidates with *CDX2* expression, suggesting that HNF4α and KLF5 may be likely CDX2 partners (Fig. 6d). Notably, CDX2 has been previously identified in GC as a driver of intestinal metaplasia^51^, and KLF5 and GATA4/6 have been previously reported as oncogenic TFs in GC that cooperate to upregulate *HNF4*α^52^.

To experimentally confirm genomic co-occupancy of CDX2 with HNF4α (the highest correlated factor), we performed CDX2 and HNF4α ChIP-seq on OCUM-1 gastric cells, and integrated the TF binding locations with predicted super-enhancer locations. In OCUM-1 cells, CDX2 and HNF4α binding summits (*q*<0.01, MACS2) exhibited high co-occurrence (500 bp window), with 76% of CDX2 binding co-occurring with HNF4α (known as CDX2/HNF4α sites) (Fig. 6e). Comparing the top 50% of high *CDX2*-expressing GCs against the lowest 50%, we found that in the former samples, recurrent somatic gain predicted super-enhancers were indeed associated with higher CDX2 binding densities (123 bindings per million base pair, Mbp versus 92 Mbp; see Methods). CDX2/HNF4α sites were preferentially localized to somatic gain predicted super-enhancers relative to unaltered predicted super-enhancers (*P*=2.4 × 10^−4^, chi-square test), and both CDX2 and HNF4α binding signals were increased at somatic gain predicted super-enhancers relative to unaltered predicted super-enhancers (Fig. 6f). Similar CDX2 and HNF4α ChIP-seq results were also obtained in SNU16 cells (Supplementary Fig. 16a). This result indicates that somatic gain predicted super-enhancers in GC are associated with CDX2 and HNF4α occupancy.

To test if CDX2 and HNF4α might play a role in GC super-enhancer maintenance, we performed silencing of each TF, either singly or both factors simultaneously, followed by genome-wide H3K27ac profiling. Depletion of either factor, either individually or in combination, did not induce global changes in H3K27ac in OCUM-1 cells (Supplementary Fig. 16b). However, *CDX2* and *HNF4*α*-*silencing led to specific H3K27ac alterations in 9.7 Mb and 4.3 Mb of the genome respectively, and double-TF knockdown induced significantly greater H3K27ac depletion (*P*=3.4 × 10^−29^ and 1.2 × 10^−88^ compared to *CDX2* and *HNF4*α*-*alone, one-sided Wilcoxon rank sum test) (Supplementary Fig. 16c). For both single-TF and double-TF silencing, H3K27ac depletion occurred more prominently at somatic gain predicted super-enhancers compared to predicted typical enhancers, suggesting a heightened sensitivity of super-enhancer activity to TF depletion (Fig. 6g, Supplementary Fig. 16d, Supplementary Data 4; *P*=5.3 × 10^−7^; *P*=1.8 × 10^−17^; *P*=1.5 × 10^−10^ for *CDX2*, *HNF4*α and *CDX2/HNF4*α respectively, one-sided Wilcoxon rank sum test). Supporting the specificity of these effects, H3K27ac depletion at predicted super-enhancers was more pronounced at regions centred at CDX2 or HNF4α binding sites, particularly at sites co-occupied by both factors (Fig. 6h). Similar results were also obtained in SNU16 cells (Supplementary Fig. 16e). Next, to assess relationships between predicted super-enhancers and gene expression, we focused on predicted super-enhancers exhibiting H3K27ac depletion after TF silencing. We observed that >60% of predicted super-enhancer target genes also exhibited reduced expression after TF silencing (si*CDX2*, *P*=4 × 10^−4^, empirical test; si*HNF4*α, *P*<1 × 10^−4^, empirical test; si(*CDX2/HNF4*α), *P*<1 × 10^−4^, empirical test; Supplementary Fig. 16f). This proportion significantly exceeded that expected by chance, as assessed by permutation analysis (Methods). Taken collectively, these results support a functional requirement for CDX2 and HNF4α in GC super-enhancer maintenance.

## Discussion

GC is a clinically heterogeneous disease, and besides surgery and chemotherapy, only traztuzumab (anti-HER2) and ramucirumab (anti-VEGFR2) are approved clinically with other molecularly targeted agents proving unsuccessful to date^20^. Epigenomic deregulation has emerged as an important pathway in gastric tumorigenesis, with chromatin modifier genes (eg, *ARID1A*) being frequently mutated in GC^53^,^54^ and epigenetic alterations associated with gastric pre-malignancy^55^. To date however, the vast majority of GC epigenomic studies have focused on promoter DNA methylation in the context of tumour suppressor gene silencing (for example, *CDH1*, *RUNX3*). In contrast, very little is currently known about distal regulatory elements (that is, enhancers) in GC.

Here, we analysed >35k predicted enhancer elements identified through micro-scale histone modification profiling of primary gastric tumours, matched non-malignant tissues and GC cell lines. Small-scale ChIP protocols are known to be technically challenging and may sometimes result in significant between-sample variability. Reassuringly, we have previously demonstrated that Nano-ChIP signals between tumours and normal samples exhibit a good concordance with orthogonal ChIP-qPCR results^4^ and in the present study we also performed extensive quality control analyses, including variations in mapping stringency, biological replicate analysis, promoter ChIP enrichment and CHANCE analysis, to confirm that the vast majority (85–100%) of our Nano-ChIPseq libraries are of acceptable quality. Our focus on recurrent epigenomic alterations present in multiple samples further ensured that our biological conclusions are likely to be robust, as shown by the observation that 84% of the recurrent somatic gain predicted super-enhancers were still rediscovered when analysis was confined to only those ‘high-quality' tumour/normal pairs passing both promoter-based and CHANCE quality analysis. Nevertheless, we also note there is still scope for improving the data quality of such experiments, such as using spike-in approaches to better ascertain inter-experimental variations or to exclude extreme experiments^56^.

Our study was motivated by recent studies in other cancer types demonstrating a fundamental role for distal enhancers and super-enhancers in cell identity and disease^7^,^11^,^16^,^25^,^45^. Indeed, in our study and others^11^, recurrent predicted super-enhancers largely manifested at known oncogenes and genes participating in oncogenic processes (Fig. 2d). We also observed high levels of enhancer variation between individual samples, exceeding proximal promoter elements (Fig. 1d). When compared against other tissues and tumour-types, almost 60% of GC predicted super-enhancers were tissue-specific (Supplementary Fig. 15). It is worth noting that in the current study we studied GCs as a general category against matched non-malignant gastric tissues for maximal sensitivity; however, recent studies have highlighted distinct histopathological and molecular GC subtypes^33^,^34^,^35^. Indeed, a preliminary analysis comparing recurrent somatic gain predicted super-enhancers between 10 intestinal and 6 diffuse GCs identified 471 and 224 predicted super-enhancers specific to intestinal- and diffuse-type GCs respectively, and 516 common predicted super-enhancers. These results also suggest that there may exist distinct enhancer alterations in different histological subtypes of GC. Such findings reflect the exquisite tissue-specific nature of enhancer elements, and the consequent need for generating comprehensive enhancer catalogues in expanded patient cohorts and in many different tumour types.

The majority of samples analysed in our study were primary tissues derived directly from patients, rather than *in vitro* cultured cell lines. By comparing predicted enhancer activities (H3K27ac) between tumours and matched normals, we were able to further sub-classify cell line predicted super-enhancers according to their somatic alteration status (somatic gain, somatic loss and unaltered). Supporting their biological distinctiveness, the subcategorized predicted super-enhancers also displayed specific differences in other orthogonal features, including epigenomic patterns (H3K4me1, DNA methylation), gene transcription and cancer hallmarks. Notably, in our data only a small fraction of somatic gain predicted super-enhancers localized to regions of copy number amplification. Our results thus complement recent studies implicating focal amplification as a mechanism for super-enhancer activity in cancer^57^. The ability to subclassify predicted super-enhancers according to *bona-fide* somatic gain or loss is likely to improve downstream attempts to pinpoint oncogenic mechanisms responsible for establishing super-enhancers in cancer. Such approaches are also possibly extendable to other disease states^45^.

*A priori* consideration of predicted super-enhancer heterogeneity may also prove useful when analysing germline variants associated with disease risk. While previous findings have reported that disease-associated SNPs are generally over-represented in regulatory elements^7^,^9^,^11^, we found that somatic altered, but not unaltered predicted super-enhancers, were specifically enriched in SNPs associated with cancer and inflammatory gastrointestinal disease (a known risk factor for gastrointestinal cancer). SNPs in these regions may alter disease risk and cancer development through several non-exclusive mechanisms, including modification of TF binding motifs^58^, regulation of long-range chromatin interactions^26^ or alteration of H3K27ac levels. Indeed, in our study, we observed that two SNPs associated with colorectal cancer (CRC) risk (rs10505477 and rs10411210)^59^ were also associated with local changes in chromatin modification in primary GCs. There are several reasons why it may be plausible to integrate CRC risk data with GC. First, at least one of these CRC risk SNPs (rs10505477) has been reported to also influence GC clinical outcome in both treatment response and patient survival^60^. Second, the key TFs associated with the GC predicted super-enhancers (*CDX2*, *HNF4*α) are also known to regulate colonic development^61^,^62^. Third, the role of intestinal metaplasia (IM) as a pre-malignant risk factor for GC is well-established, and in IM the gastric epithelial cells adopt a cellular architecture and appearance similar to colonic epithelium^20^. The observation that these genetic variants, while present in germline DNA, may influence chromatin structure and gene expression in the tumour has also been observed in CRC^63^. These results further highlight the importance of studying aberrant epigenetic states to refine our understanding of germline processes underlying disease predisposition.

Our results suggest certain general principles regarding how individual super-enhancers in GC might interact with the *cis-* and *trans-*acting transcriptional machinery. Using two distinct long-range chromatin interaction assays (Capture-C and 4C), we observed several examples of somatic gain predicted super-enhancers engaging both proximal and distal genes exhibiting elevated tumour expression. It has been proposed that genes linked to somatic gain predicted super-enhancers are likely to occupy similar topological associating domains, established through cohesin-mediated enhancer-promoter loops^64^. The ability of somatic gain predicted super-enhancers to influence both proximal and distal gene expression implicates predicted super-enhancers as pivotal regulators of aberrant gene expression in gastric tumours, which can contribute to disease progression and chemoresponse^35^ (Fig. 5b). At the *trans*- level, our data revealed that somatic gain predicted super-enhancers in GC are associated with CDX2 and HNF4α occupancy. Previous studies have shown that aberrant CDX2 expression in the stomach is associated with intestinal metaplasia of the mucosal epithelial cells, an important early event in gastric tumour formation and that CDX2 has the potential to function as a GC oncogene^65^. HNF4α has also been recently implicated in GC, as a target of both the lineage-specific oncogenes KLF5 and GATA factors^52^, and the AMPK signalling pathway^20^. Our results in primary human tumours are supported by recent findings in the mouse small intestine, where CDX2 has been found to regulate HNF4α occupancy to control intestinal gene expression^66^. Echoing these studies, we also found that CDX2/HNF4α depletion effected chromatin alterations at local regions concentrated at CDX2 and/or HNF4α binding sites. A potential limitation in our study is that the modest albeit significant differences in H3K27ac observed after TF silencing may be due to use of transient knockdown compared to stable gene silencing. While it is thus possible that CDX2 and HNF4α may influence GC super-enhancer activity in a more wide-spread manner, these results nevertheless support a specific role for CDX2 and HNF4α in GC predicted super-enhancer maintenance.

In conclusion, our study demonstrates a role for heterogeneity in predicted super-enhancers and the utility of intersecting chromatin profiles from primary tissues and cell lines to dissect regulatory biology. This first-generation roadmap of GC distal enhancers now renders possible future integrative studies involving transcriptional features associated with GC predicted enhancers (eRNAs), and identifying somatic regulatory mutations perturbing predicted super-enhancer activity^67^. We also note that in this study, we have focused on heterogeneity between patients; however, intra-tumour heterogeneity is also emerging as an important mechanism of GC treatment resistance^68^. Future studies should be focused on investigating the role of epigenetic deregulation in GC intra-tumour heterogeneity. Taken collectively, these studies will likely deepen our knowledge of GC enhancer biology and regulatory circuits underlying this deadly malignancy.

## Methods

### Primary tissue samples and cell lines

Primary patient samples were obtained from the SingHealth tissue repository with approvals from the SingHealth Centralised Institutional Review Board and signed patient informed consent. ‘Normal' (that is, non-malignant) samples used in this study refers to samples harvested from the stomach, from sites distant from the tumour and exhibiting no visible evidence of tumour or intestinal metaplasia/dysplasia upon surgical assessment. Tumour samples were confirmed by cryosectioning to contain >40% tumour cells. FU97, MKN7, OCUM-1 and RERF-GC-1B cell lines were obtained from the Japan Health Science Research Resource Bank. KATO-III and SNU16 cells were obtained from the American Type Culture Collection. NCC-59 was obtained from the Korean Cell Line Bank. YCC3, YCC7, YCC21, YCC22 were gifts from Yonsei Cancer Centre, South Korea. Cell line identities were confirmed by STR DNA profiling performed at the Centre for Translational Research and Diagnostics (Cancer Science Institute of Singapore, Singapore). STR profiles were assessed according to the standard ANSI/ATCC ASN-0002-2011 nomenclature, and the profiles of our cell lines showed >80% similarity to the reference databases. MKN7 cells—one commonly misidentified line by ICLAC (http://iclac.org/databases/cross-contaminations/) was confirmed by showing a perfect match (100%) with the MKN7 reference profile in the Japanese Collection of Research Bioresources Cell Bank. MycoAlert Mycoplasma Detection Kits (Lonza) and MycoSensor qPCR Assay Kits (Agilent Technologies) were used to detect mycoplasma contamination. All cell lines were negative for mycoplasma contamination. For this study, we selected OCUM-1 and SNU16 cells as our main cell line models for two reasons. First, OCUM-1 and SNU16 cells were originally isolated from patients with poorly differentiated GC, and the majority of primary GCs in our study are poorly differentiated (63%). Second, OCUM-1 and SNU16 have been previously used as GC models in many other published studies^69^,^70^, and are thus regarded as accepted GC models in the field. Thus, OCUM-1 and SNU16 were used as consistent cell line models for several experiments, including Capture-C, 4C, enhancer CRISPR, TF binding and TF knockdown.

### Nano ChIPseq

Nano-ChIPseq was performed as described^4^ with slight modifications. For primary tissues, fresh-frozen cancer and normal tissues were dissected using a razor blade in liquid nitrogen to obtain ∼5 mg sized piece for each ChIP. Tissue pieces were fixed in 1% formaldehyde/PBS buffer for 10 min at room temperature. Fixation was stopped by addition of glycine to a final concentration of 125 mM. Tissue pieces were washed three times with tris buffered saline with EDTA (TBSE) buffer. For cell lines, 1 million fresh harvested cells were fixed in 1% formaldehyde/medium buffer for 10 min at room temperature. Fixation was stopped by addition of glycine to a final concentration of 125 mM. Fixed cells were washed three times with TBSE buffer, and centrifuged (5000, r.p.m., 5 min). Pelleted cells and pulverized tissues were lysed in 100 μl 1% SDS lysis buffer and sonicated to 300–500 bp using a Bioruptor (Diagenode). ChIP was performed using the following antibodies: H3K4me3 (07-473, Millipore); H3K4me1 (ab8895, Abcam); H3K27ac (ab4729, Abcam).

After recovery of ChIP and input DNA, whole-genome-amplification was performed using the WGA4 kit (Sigma-Aldrich) and BpmI-WGA primers. Amplified DNAs were purified using PCR purification columns (QIAGEN) and digested with BpmI (New England Biolabs) to remove WGA adapters. Thirty nanograms of amplified DNA was used for each sequencing library preparation (New England Biolabs). Eight libraries were multiplexed (New England Biolabs) and sequenced on two lanes of Hiseq2500 (Illumina) to an average depth of 20–30 million reads per library.

### Sequence mapping and ChIP-seq density analysis

Sequence reads were mapped against human reference genome (hg19) using Burrows-Wheeler Aligner (BWA-MEM, version 0.7.0), after trimming the first and the last ten bases prior to alignment. Only high-quality mapped reads (MAPQ≥10) were retained for downstream analyses. The MAPQ value (≥10) was chosen as this has (i) been previously reported to be a good value to use for good/confident read mapping^71^; (ii) MAPQ≥10 has also been indicated by the developers of the BWA-algorithm to be a suitable threshold to use for confident mappings using their software^72^; and (iii) studies assessing various algorithms for read alignment have also shown that mapping quality scores do not correlate well with the likelihood of read mapping being true/accurate and have shown that the level of accuracy obtained for mapping accuracy plateaus between a 10–12 MAPQ threshold^73^. We also note that in our study we have focused on *recurrent* predicted enhancers and super-enhancers that are reliably detected in multiple samples, which increases the robustness of our analysis. Sequencing coverage was computed using MEDIPS with a 50 bp window size and read length extension to 200 bp. Peaks with significant ChIP enrichment (FDR<5%) relative to input libraries were detected using CCAT (version 3). Peak densities within a region were computed by counting the total number of mapped reads normalized by the library and region size, a metric equivalent to reads per million mapped reads per kilobases (RPKM). This normalization method adjusts for biases due to the higher probability of reads falling into longer regions and has been applied in previous studies^74^. We thus elected to apply RPKM-based normalization to make our study comparable to these other studies. To account for background signals, read densities of each ChIP library were corrected against the corresponding input library. Read densities across samples were corrected for potential batch effects (for example, date of ChIP assay) using COMBAT and to ensure equal sample variation. Of 17,360 recurrent predicted enhancers detected in two or more cell lines, 98% were present in at least one primary sample (normal or GC).

### Quality control assessment of nano-ChIPseq data

We assessed qualities of the ChIP libraries (H3K27ac, H3K4me3 and H3K4me1) using two different methods. First, we estimated ChIP qualities, particularly H3K27ac and H3K4me3, by interrogating their enrichment levels at annotated promoters of protein-coding genes. Specifically, we computed median read densities of input and input-corrected ChIP signals at 1,000 promoters associated with highly expressed protein-coding genes. For each sample, we then compared read density ratios of H3K27ac over input as a surrogate of data quality, retaining only those samples where the H3K27ac/input ratio was greater than four-fold. Using these criteria, 48 out of 50 H3K27ac samples (GC lines and primary samples) exhibited greater than four-fold enrichment, indicating successful enrichment. A similar analysis was also performed for the H3K4me3 libraries (a promoter mark), and all 42 libraries satisfied this quality control criteria. Second, we used CHANCE (CHip-seq ANalytics and Confidence Estimation)^22^, a software for ChIP-seq quality control and protocol optimization that indicates whether a library shows successful or weak enrichment. We found that the large majority (85%) of samples in our study exhibited successful enrichment as assessed by CHANCE. The assessment status for each library, as assessed by both methods, are reported in Supplementary Data 1.

We experimentally generated a second biological replicate of H3K27ac Nano-ChIP-seq using KATO-III cells, and also compared our results against independent H3K27ac KATO-III data generated from regular ChIP-seq protocols^23^. The published sequencing reads were processed similarly to our NanoChIP-seq libraries, excluding sequence trimming. Peaks detected by CCAT at a FDR <5% were compared.

### Chromatin accessibility and binding enrichment

Chromatin accessibility profiles of Epigenome Roadmap normal gastric tissues were obtained from the Gene Expression Omnibus (GSM1027325, GSM1027320)^28^. Read densities of chromatin accessibility profiles were computed for predicted enhancer regions and compared against 100,000 randomly selected regions in RPKM units. We also computed fractions of predicted enhancers overlapping open chromatin regions (.narrowPeak) and active regulatory elements (H3K27ac, .gappedPeak) from 25 Roadmap chromatin accessibility and H3K27ac profiles. For TF binding enrichment analysis, P300 and other TF binding coordinates curated by the ENCODE (wgEncodeRegTfbsClusteredV3.bed), were downloaded from the UCSC genome browser. Overlaps of at least 1 bp were identified using BEDTools intersect. Levels of evolutionary sequence conservation were assessed using PhastConst scores (Castelo R. phastCons100way.UCSC.hg19: UCSC phastCons conservation scores for hg19. R package version 3.2.0). The maximum score within 500 bp from the enhancer midpoint was used as the enhancer conservation score^75^. Conservation scores were also computed for 10,000 randomly selected regions, excluding pre-detected enhancer regions.

### Identification of predicted super-enhancers

Predicted enhancers were defined as enriched H3K27ac regions at least 2.5 kb from annotated TSS and also showing enrichment of H3K4me1 and depletion of H3K4me3 (ref. 6). TSS annotations for this study were derived from GENCODE version 19. H3K4me3/H3K4me1 log ratios were computed using aggregated H3K4me3 and H3K4me1 signals from GC cell lines and primary samples. Distal predicted enhancers exhibiting high H3K27ac signals, but exhibiting high H3K4me3/H3K4me1 log ratios (>2.4, ref. 26) were classified as mistaken predictions and thus excluded from analyses. Predicted enhancers were then further subdivided into predicted super-enhancers or typical enhancers using the ROSE algorithm. Predicted super-enhancer regions with at least one base overlap across multiple GC lines were merged using BEDTools, and predicted enhancers localizing to regions distinct from the predicted super-enhancer regions were termed predicted typical enhancers. The presence of predicted typical or predicted super-enhancers in individual samples was determined by the level of H3K27ac enrichment above background (*P*<0.01, empirical test), the latter being the H3K27ac signal (in RPKM) from 100,000 randomly selected regions. To assign predicted enhancers/super-enhancers to genes, we calculated distances from the predicted enhancer/super-enhancer centre to the nearest active TSS, defined as a promoter (500 bp flanking at TSS) with H3K27ac enrichment above randomly chosen regions^11^. Genes associated with recurrent predicted super-enhancers were tested for oncogene enrichment using a one-sided Fisher's exact test. The top 500 oncogenes^76^ were used. To identify recurrent predicted enhancer and predicted super-enhancers, the regions in each GC line were ranked according to signal strength. The ranks of each predicted enhancer/super-enhancer across the lines were multiplied to compute the rank product. To determine the statistical significance of the rank product, we compared the observed rank product against a null distribution—ranks in each line were reshuffled and the rank products computed. The reshuffling procedure was repeated for 10,000 iterations. Observed rank products less than the null distribution were considered statistically significant.

### Validation of predicted interactions

Super-enhancer/gene assignments were validated using three orthogonal interaction data sets. These included:

- Predetermined interactions detected by PreSTIGE^30^ from 12 cell lines. PreSTIGE interaction data were downloaded from the PreSTIGE website (prestige.case.edu), involving *cis*-regulatory elements and target genes.
- *cis*-regulatory elements/gene assignment by GREAT^31^ using the default parameters.
- Reference sets of enhancer-promoter interactions from RNAPII ChIA-PET studies in K562, HCT-116, NB4, MCF-7, HeLa-S3 and GM12878 cells. ChIA-PET interaction data were downloaded from encodeproject.org and GSE72816. All interactions identified in each biological replicate were considered for validation. These interactions involved two loci (anchors), one of which is within 2.5 kb of a TSS and the other anchor overlapping predicted super-enhancer regions found in our study.

Besides (i)-(iii), additional validation was performed using Capture-C analysis on GC lines (see Fig. 4 of the Main Text).

### Functional enrichment analysis

We used GOrilla^32^ to identify biological processes (Gene Ontology annotations) enriched in recurrent predicted super-enhancer/gene promoter or predicted typical enhancer/gene promoter interactions. Default GOrilla parameters were used, and genes from GENCODE v19 were used as background. To ensure comparability, predicted typical enhancers with the highest H3K27ac across cell lines were selected to match the same number of recurrent predicted super-enhancers. To select the former, predicted typical enhancers were ranked in each line and were chosen based on the rank product score. The most significant terms (> 1.5-fold enrichment) associated with the recurrent predicted super-enhancers were then compared against enrichment levels associated with the top predicted typical enhancers. Besides GOrilla, we also studied functional enrichments associated with recurrent predicted super-enhancers and top predicted typical enhancers using GREAT using default parameters, as GREAT provides correction against genes flanked by larger intergenic regions^31^. Significant terms (also with >1.5-fold enrichment) were ordered based on Binomial *P*-values.

### Cell-line-derived super-enhancers in primary samples

Regions showing H3K27ac enrichment or depletion of by two-fold or greater and with absolute differences of greater than 0.5 RPKM^4^ were considered differentially present between GCs and matched normal samples. For PCA, we used signals from predicted super-enhancers showing somatic gain in two or more patients. PCA analysis was performed using R and plotted using the ‘pca3d' package. We estimated the required sample size to achieve 80% power and 5% type I error (http://powerandsamplesize.com/) based on the average signals of 100 predicted super-enhancers (Supplementary Data 2) from tumour and normal samples. This result yielded a recommended sample size of 13 (average), which is met in our study (19N/T). Three classes of predicted super-enhancers were defined based on the primary samples: (i) somatic gain, (ii) somatic loss and (iii) unaltered. Genes associated with (i), (ii) and (iii) were mapped to gene groups previously reported in Hnisz, 2013 (ref. 11), where each group is a compilation of several gene ontology categories and used as a proxy for various cancerous hallmarks^11^. Statistical significance was computed using one-sided Fisher's exact test in R. To assess lineage-specificities of the recurrently gained somatic predicted super-enhancers across different tissue types, we computed overlaps between the gastric predicted super-enhancers against other non-gastric tissues^11^. An enrichment ratio with each non-gastric tissue was computed based on the total observed overlap versus the total overlap by chance.

### Capture-C and data analysis

Capture-C was performed as previously described^36^. Briefly, 1 × 10^7^ cells were crosslinked by 2% formaldehyde, followed by lysis, homogenization, DpnII digestion, ligation and de-crosslinking. DNA was sonicated using a Covaris to 150–200 bp to produce DNA suitable for oligo capture. Three micrograms of sheared DNA was used for sequencing library preparation (New England Biolabs). Predicted super-enhancer sequences were double captured by sequential hybridization to customized biotinylated oligos (IDT, Supplementary Data 5) and enrichment with Dynabeads (LifeTech). Captured DNA was sequenced on an Illumina MiSEQ using the 150 bp paired-end configuration.

Preprocessing of raw reads was performed to remove adaptor sequences (trim_galore, http://www.bioinformatics.babraham.ac.uk/projects/trim_galore/) and overlapping reads were merged using FLASH. In order to achieve short read mapping to the hg19 reference genome, the resulting preprocessed reads were then *in-silico* digested with DpnII and aligned using Bowtie (using p1, m2, best and strata settings). Aligned reads were processed using Capture-C analyser^36^ to (i) remove PCR duplicates, and (ii) classify subfragments as ‘capture' if they were contained within the capture fragment; ‘proximity exclusion' if they were within 1 kb on either side of the capture fragment; or ‘reporter' if they were outside of the ‘capture' and ‘proximity exclusion' regions. We additionally used the r3Cseq package^37^ on the capture and reporter fragments to identify significant interactions of the viewpoint against a scaled background (*Q*<0.05, FDR) and also to compare interaction profiles between different cell lines.

### 4C-seq and data analysis

4C templates were prepared using previously published protocols with slight modifications^77^. In brief, cultured cells were diluted into single-cell suspensions, and chromatin was cross-linked with 1% formaldehyde for 10 min at room temperature. Cells were lysed and cross-linked DNA was digested with the primary restriction enzyme HindIII-HF [R3104L, New England Biolabs (NEB)]. Next, HindIII-digested DNA was subjected to proximity ligation using T4 DNA ligase (EL0013, Thermo Scientific), followed by cross-link removal using Proteinase K (AM2546, Ambion), yielding 3C libraries. The 3C libraries were then subjected to a second restriction enzyme digestion using DpnII (R0543L, NEB), followed by a circularization reaction using T4 DNA ligase. For each viewpoint, 3.2 μg of the resulting 4C templates was used to perform a scale-up inverse, nested PCR (Supplementary Table 5), of which 32 reactions (100 ng in each) were pooled and purified using the MinElute PCR Purification kit (Qiagen). Ten micrograms of the PCR products were then run on 4–20% TBE PAGE gels (5 μg per well). On the gel, smears from 200 to 600 bp were excised and unwanted PCR product bands were removed. DNA was then extracted from the cut-out gel pieces for next-generation sequencing on an Illumina Miseq (2 × 250 bp).

Inverse primers were designed based on a viewpoint concept. The UCSC Genome Browser (assembly: Feb. 2009 (GRCh37/hg19)) was used to locate the region of interest. Upon addition of HindIII and DpnII tracks, two HindIII restriction sites flanking the region of interest were identified and the sequence between the nearest HindIII and DpnII restriction sites were selected as the viewpoint region. Based on this region, two pairs of primers (outer and nested) were designed using the Primer-BLAST program (National Center for Biotechnology Information (NCBI)) with the following adaptations to the default settings: optimal primer melting temperature of 58 °C, with a minimum of 55 °C and maximum of 60 °C; GC content between 39 and 60%. Appropriate adaptors (Nextera Index Kit—PCR primer, Nextera transposase sequence) and index sequences were then added to the nested primer pair. Outer and nested primers used in this study are presented in Supplementary Tables 4 and 5 respectively.

Primer sequences at the 5′ ends of sequencing reads were trimmed using TagDust2, and mapped to the reference genome (hg19) using Bowtie2 (2.2.6). Unaligned reads were trimmed at the first 50 base pairs before realigning them to the reference genome. Only uniquely mapped reads with MAPQ≥30 were used in downstream analyses. Statistical significant interactions (*Q*<0.05, FDR) were detected using r3Cseq using a non-overlapping window approach (window size=5 kb). Signal plots of 4C data were generated using Basic4CSeq. Detected interactions within DNA amplified regions were excluded. Interactions were then mapped to genes, using promoters (+/− 2.5 kb from annotated TSSs from GENCODE v19) overlapping with the interactions.

### CRISPR/Cas9 enhancer deletions

CRISPR sgRNA target search was performed using online software created by the Feng Zhang laboratory (http://tools.genome-engineering.org). sgRNA pairs were designed to target sequences flanking enhancers identified for deletion. Briefly, sequences corresponding to 100 bp upstream/20 bp downstream of the 5′ end of the enhancer, and sequences corresponding to 20 bp upstream/100 bp downstream of the 3′ end of the enhancer, were used for the search. Top hits with the lowest level of coding region off-target predictions were chosen. sgRNAs were cloned into the pSpCas9(BB)-2A-GFP or -Puro vectors (Addgene). Briefly, pairs of oligonucleotides were designed and procured from Integrated DNA Technologies, Inc. for each CRISPR target. Oligonucleotide pairs were then annealed to form DNA duplexes containing overhangs on both sides for ease of cloning. Guide RNAs used to target 5′ ends of individual enhancers were cloned into Bbs I-digested pSpCas9(BB)-2A-GFP vectors, while sgRNAs targeting 3′ ends of each enhancer were cloned into Bbs I-digested pSpCas9(BB)-2A-Puro vectors. The inserts and vectors were ligated using T4 DNA Ligase (New England Biolabs). DH5α cells were transformed with the ligation product and plated on LB agar supplemented with ampicillin. Colonies were picked and cultured, and plasmids extracted using the Wizard Plus SV Minipreps DNA Purification System (Promega). Sequences of plasmids were confirmed by performing Sanger sequencing. Oligonucleotides used for these experiments are listed in Supplementary Table 6.

SNU16 and OCUM-1 cells were grown to 80–90% confluence in Roswell Park Memorial Institute (RPMI) supplemented with 10% fetal bovine serum (FBS), 1xP/S and 0.5XNEAA. Cells were harvested and spun down, treated with Typsin for 5 min at 37 degrees, and re-suspended by pipetting to achieve single cell suspensions. Cell numbers were counted, and cells were washed once with 1 × PBS before resuspension in Resuspension buffer (R) at 1 × 10^7^ cells/ml. For every 1 × 10^7^ cells in 1 ml of Resuspension buffer, 25 μg of pCas9-GFP-sgRNA and 25 μg of pCas9-Puro-sgRNA plasmid were mixed with SNU16 or OCUM-1 cells. Hundred microlitres of each cell suspension was electroporated using a 100 μl Neon pipette in a Neon tube containing 3 ml of Electrolytic Buffer (E2). Electroporation conditions were: Pulse, V 1050, MS 30, Number 2. After electroporation, cells were plated onto 8 ml of RPMI supplemented with 10% FBS, 1xP/S and 0.5XNEAA. At 24 h after initial transfection, the cells were treated with 10 μg of Puromycin for 48 h, and the remaining GFP-positive cells were sorted using FACS. The remaining surviving cells (both GFP-positive and Puromycin-resistant) were then subsequently analysed using qPCR to estimate knockout efficiencies.

We performed quantitative PCR (qPCR) to determine the efficiency of deletion of individual enhancers in CRISPR/Cas9-targeted cells. Genomic DNA of targeted and untargeted cells (pooled) was extracted using the AllPrep DNA Micro Kit (QIAGEN) and subjected to qPCR in technical triplicates using KAPA SYBR FAST qPCR Master Mix (Kapa Biosystems) on a CFX96 Touch Real-Time PCR Detection System (Bio-Rad Laboratories, Inc.). Primers used in these reactions are listed in Supplementary Table 6 (primers with ‘Int' in their names were used for this purpose). Relative amount of the specific targeted region present in the genomic DNA samples was calculated using the comparative C_T_ (ΔΔC_T_) method, normalized to the *GAPDH* gene and relative to untargeted cells.

Genomic DNA was extracted from the sorted cells using a previously described protocol^78^. Briefly, cells were triturated in 0.5 × Direct-Lyse buffer (10 mM Tris pH 8.0, 2.5 mM EDTA, 0.2 M NaCl, 0.15% SDS, 0.3% Tween-20) and subjected to the following heating and cooling program: 65 °C for 30 s, 8 °C for 30 s, 65 °C for 1.5 min, 97 °C for 3 min, 8 °C for 1 min, 65 °C for 3 min, 97 °C for 1 min, 65 °C for 1 min and 80 °C for 10 min. Subsequently, the lysates were diluted approximately 4 × in water and 3 μl of the diluted lysates were used to perform 20-μl PCR reactions using Taq DNA Polymerase (Life Technologies). Primers used are in Supplementary Table 6 (primer pairs of ‘5′ F' and ‘3′ R' for each enhancer).

*RT-qPCR to measure gene expression levels*. Cells were FACS sorted for GFP-positive cells and total RNA was extracted from cells using AllPrep DNA/RNA Micro Kit (QIAGEN). Reverse transcription was performed on pooled cells using iScript Select cDNA Synthesis Kit (Bio-Rad) with random primers. qPCR was conducted using TaqMan Gene Expression Master Mix and TaqMan probes (Applied Biosystems) on a CFX384 Touch Real-Time PCR Detection System (Bio-Rad). All qPCR experiments were run in triplicate, and mean values were used to determine mRNA levels. Relative quantification was performed using the comparative C_T_ method with *GAPDH* as the reference gene and with the formula 2-ΔΔC_T_.

### Copy number alterations and DNA methylation

Genomic DNAs from gastric tumours and matched normal gastric tissues were hybridized on Affymetrix SNP6.0 arrays (Affymetrix, Santa Clara, CA). Data in CEL format was processed in the following order: (1) Normalization: Raw.CEL files were processed using Affymetrix Genotyping Console 4.2. Reference models were created from SNP6.0 profiles of normal gastric tissues according to the hybridization batch. Copy number changes in cell lines and primary tumour samples were determined by using the reference model from primary normal samples. (2) Segmentation: Copy number segmentation data were produced using the circular binary segmentation (CBS) algorithm implemented in the DNAcopy R package. The *P*-value cutoff for detecting a change-point was 0.01, with a permutation number of 10,000. Copy number gain and loss regions were defined for showing average log ratios of >0.6 and<−1.0, respectively. Illumina HumanMethylation450 (HM450) Infinium DNA methylation arrays were also used to assay DNA methylation levels. Methylation β-values were calculated and background corrected using the methylumi R BioConductor package. Normalization was performed using the BMIQ method (watermelon package in R).

### RNAseq and analysis

Total RNA was extracted using the Qiagen RNeasy Mini kit. RNA-seq libraries were constructed according to manufacturer's instructions using Illumina Stranded Total RNA Sample Prep Kit v2 (Illumina, San Diego, CA) Ribo-Zero Gold option (Epicentre, Madison, WI) and 1 μg total RNA. Completed libraries were validated with an Agilent Bioanalyzer (Agilent Technologies, Palo Alto, CA) and applied to an Illumina flow cell via the Illumina Cluster Station. Sequencing was performed using the paired-end 101 bp read option. RNA-seq reads were aligned to the human genome (hg19) using TopHat2-2.0.12 (default parameter and --library-type fr-firststrand). The per base sequence quality and per sequence quality scores of the mapped reads was assessed using FastQC version 0.10.1 (http://www.bioinformatics.babraham.ac.uk/projects/fastqc/). Transcript abundances at the gene level were estimated by cufflinks. Gene expression from primary samples showing variation greater than zero was corrected for potential batch effects using ComBat. Gene expression values were measured in FPKM units. Differential expression between groups was identified as genes showing altered expression by at least two-fold and absolute differences of 0.5 FPKM.

### Survival analysis

GC samples from seven independent studies were clustered using a K-medoids approach. Only genes with expression values in all seven studies were used in analyses. Kaplan–Meier survival analysis was employed with overall survival as the outcome metric. Log-rank tests were used to assess the significance of Kaplan–Meier curves. Multivariate analysis involving additional variables, such as age, tumour stage, Lauren's histological subtypes and locality (Asian versus Non-Asian), was performed using Cox regression.

### Disease-associated SNP analysis

Trait-associated SNPs were downloaded from the UCSC browser of GWAS (27 August 2015). For this study, we focused on SNPs occurring in noncoding regions and excluded SNPs within coding regions. We computed overlaps between SNPs from each trait/disease and somatic predicted super-enhancers using BEDtools ‘intersect' (nGWAS), and compared nGWAS against the total number of disease-associated SNPs outside the predicted super-enhancers (nGWAS'). As an additional control, we created an ‘SNP background' model using a set of all SNPs from two commonly used SNP arrays (Illumina HumanHap550 and Affymetrix SNP6). The number of SNPs from the SNP background overlapping the predicted super-enhancers was calculated (nBackground) and compared against the total number of background SNPs outside the predicted super-enhancers (nBackground′). The ratio of normal SNPs in predicted super-enhancers was computed as nBackground/nBackground′. Expecting that the increased number of disease-associated SNPs in predicted super-enhancers is associated with a high prevalence of SNPs in these regions, our null hypothesis is therefore that there is no difference between the ratio of disease-associated SNPs and the ratio of normal SNPs (enrichment ratio). Chi-square tests were conducted, with enrichment *P*-values of<0.01 considered statistically significant. To understand the relationship between risk-associated SNPs and histone modification, we identified validated SNPs in gastrointestinal diseases (for example, ulcerative colitis and colorectal cancer) found to be associated with disease in at least two independent studies. Samples were classified into two groups based on the presence of the disease-associated SNPs, using GATK Unified Genotyper. Differences of H3K27ac signals between tumour and matched normal in samples with or without disease-associated SNPs were compared.

### TF binding motif analysis

We interrogated enrichments of TFs in somatic gain predicted super-enhancers and unaltered predicted super-enhancers using the ReMap database^48^. Transcription factor binding sites with at least 60% of overlap with predicted super-enhancers were counted, and the ranks of the top 10 most enriched TFs compared. Binding densities of TFs were computed as the total binding sites detected in the regions divided by the total size of the regions in unit of million base pairs (Mbp). For CDX2, we examined CDX2 binding sites in recurrently gained somatic predicted super-enhancers to predict nearby binding of other TFs, using HOMER with default parameters^49^. The top 20 TF identified from the HOMER outputs were used for expression correlation analysis. Additionally, we also identified CDX2 co-binding motifs using PScanChIP^50^ with JASPAR 2016. Expression correlations (Spearman's correlation) between CDX2 and potential co-binding partners were evaluated.

### siRNA transfection

ON-TARGETplus Human siRNA SMARTpools (*HNF4α* and *CDX2)*, individual ON-TARGETplus Human individual siRNAs (*HNF4α)* and ON-TARGETplus Non-targeting siRNA controls (Dharmacon/Thermo Fisher Scientific) were used to transfect cells (2 × 10^5^) at 50 nM in 6-well plate, using Dharmafect 1 transfect reagent, according to the manufacturer instructions. Knockdown efficiency after 72 h RNAi treatment was examined using quantitative RT-PCR and/or Western Blot analysis (Supplementary Fig. 17).

### Western blotting

Cells (2 × 10^5^) were harvested in RIPA buffer (Sigma) and lysed for 10 min on ice. Concentration of supernatants was measured using Pierce BCA protein assay (Thermo Scientific). CDX2 (1:500; MU392A-UC, Biogenex), HNF4α (1:1,000; sc-8987, Santa Cruz Biotechnology) and GAPDH (1:3,000; 60,004-1-Ig, Proteintech Group) antibodies were used to probe the lysate.

### Quantitative RT-PCR

Total RNA was isolated using RNeasy Mini Kit (Qiagen), and DNA was removed using RNase-Free DNase Set (Qiagen). Two micrograms RNA was reverse transcribed using Superscript III First Strand Synthesis System (Invitrogen), and complementary DNA was amplified using SYBRGreen PCR Master Mix (Applied Biosystems). Fold changes were normalized to *GAPDH*. Primer sequences are as follows: HNF4α: F1-5′ GTGCGGAAGAACCACATGTACTC 3′, R1-5′ CGGAAGCATTTCTTGAGCCTG 3′, F2-5′ CTGCAGGCTCAAGAAATGCTT 3′, R2-5′ TCATTCTGGACGGCTTCCTT 3′, F3-5′ TGTCCCGACAGATCACCTC 3′, R3-5′ CACTCAACGAGAACCAGCAG 3′; CDX2: F1-5′ GCAGCCAAGTGAAAACCAGG 3′, R1-5′ CCTCCGGATGGTGATGTAGC 3′, F2-5′ AGTCGCTACATCACCATCCG 3′, R2-5′ TTCCTCTCCTTTGCTCTGCG 3′; GAPDH: F-5′ CCAGGGCTGCTTTTAACTC 3′, R-5′ GCTCCCCCCTGCAAATGA 3′.

### CDX2 and HNF4α ChIP-seq and analysis

Cells were cross-linked with 1% formaldehyde for 10 min at room temperature, and stopped by adding glycine to a final concentration of 0.2 M. Chromatin was extracted and sonicated to 500 bp. CDX2 (MU392A-UC, Biogenex) and HNF4α (sc-8987, Santa Cruz Biotechnology) antibodies were used for chromatin immunoprecipitation (ChIP). ChIPed DNA (10 ng) was used for ChIP with DNA sequencing (ChIP-seq) library construction following manufacturer protocols (New England Biolabs). Input DNA from cells prior to immunoprecipitation was used to normalize ChIP-seq peak calling. Prior to sequencing, qPCR was used to verify that positive and negative control ChIP regions amplified in the linear range. Size distributions of the library samples were checked using a Bio-analyzer (Agilent Technologies). In an initial analysis comparing recurrently gained predicted super-enhancers specific to intestinal and diffuse-type GCs (10 intestinal, 6 diffuse), we did not observe any significant differences in CDX2 binding between the two subtypes. A deeper analysis revealed, however, high within-subtype variability in *CDX2* expression between individual tumours of the same subtype, consistent with previous reports that CDX2 expression is not absolutely associated with intestinal subtype GC^79^. We thus performed a complementary analysis where the GCs were ordered by their individual *CDX2* expression levels and examined. CDX2 binding density were then computed at recurrent somatic gain predicted super-enhancers identified in GC samples showing high (*n*=8) and low (*n*=8) *CDX2* expression. In differential binding signal analysis, we computed binding signals for CDX2 and HNF4α for 200 bins spanning those predicted super-enhancers showing somatic gain or no alteration in primary samples and also detected in OCUM-1 or SNU16 cell lines. Signals were measured in RPKM units. To estimate the effect of TF knockdown on H3K27ac strength, we defined an internal control comprising observed variation of H3K27ac signals between independent wild-type (WT) samples. We then measured differences between WT samples against TF-silenced (siCDX2, siHNF4α, or double TF) samples, which were then compared against this background variation. Subregions with differences>99% of background variation were termed as H3K27ac depletion; while differences<1% of the background variation were termed as H3K27ac gain. Statistical enrichments of H3K27ac-depleted subregions corresponding to predicted super-enhancers were conducted using one-sided Fisher's exact test. To study relationships between the differential regions and their distances to nearby CDX2/HNF4α binding sites, the regions were also segregated into three categories (near, moderate, distal) based on their distance distribution. Mid-point locations between the CDX2 and HNF4α summits were used to analyse distances between H3K27ac-depleted subregions and CDX2-HNF4α cobinding sites. To study associations between gene expression and somatic gain predicted super-enhancers in TF-silenced cells, we selected genes linked to predicted super-enhancers exhibiting significant positive expression correlations with H3K27ac predicted super-enhancer signals in primary samples (*r*>0.4; *P*<0.05, two-sided *t*-test) and also observed in the GC cell lines. To assess the significance of TF knockdown on predicted super-enhancer target gene expression, we used a permutation approach. Specifically, focusing on predicted super-enhancers exhibiting H3K27ac depletion after TF silencing, we permuted the actual super-enhancer to gene assignments 10,000 times. An empirical *P*-value was then derived by counting the number of times the number of downregulated genes in the permuted gene/super-enhancer set exceeded the experimentally observed number of downregulated genes in the actual gene/super-enhancer set.

### Data availability

Histone NanoChIP-seq (GSE76153 and GSE75898), SNP array (GSE85466), RNA-seq (GSE85465) and DNA methylation data (GSE85464) generated during this study have been deposited in Gene Expression Omnibus. Previously deposited histone ChIP-seq (GSE51776 and GSE75595) and SNP array (GSE31168 and GSE36138) data that are used in this study are available in Gene Expression Omnibus. Chromatin accessibility profiles of normal gastric tissues from Epigenome Roadmap were obtained from the Gene Expression Omnibus (GSM1027325, GSM1027320). RNAPII ChIA-PET data analysed in this study was obtained from *encodeproject.org* and Gene Expression Omnibus (GSE72816). The authors declare that all other data are available within the Article or associated Supplementary information files, or available from the author on request.

## Additional information

**How to cite this article:** Ooi W. F. *et al*. Epigenomic profiling of primary gastric adenocarcinoma reveals super-enhancer heterogeneity. *Nat. Commun.* 7:12983 doi: 10.1038/ncomms12983 (2016).

## Supplementary Material

Supplementary Information Supplementary Figures 1-17, Supplementary Tables 1-6, Supplementary Discussion and Supplementary References

Supplementary Data 1 Mapping statistics and quality assessment of histone ChIP-seq libraries.

Supplementary Data 2 Top 100 super-enhancers showing somatic gain in 10 or more patients and the assigned genes.

Supplementary Data 3 CDX2 candidate binding partners using PScanChIP and their expression correlations with CDX2 expression.

Supplementary Data 4 Somatic gain predicted super-enhancers in OCUM1. a. Enrichment significance of sub-regions with depleted H3K27ac signals in predicted super-enhancers after CDX2 silencing.

Supplementary Data 5 Capture point coordinates and sequences used in Capture-C technology.

## References

[b1] Zhang L. . Gene expression profiles in normal and cancer cells. Science 276, 1268–1272 (1997). 9157888 10.1126/science.276.5316.1268

[b2] Adler A. S. . Genetic regulators of large-scale transcriptional signatures in cancer. Nat. Genet. 38, 421–430 (2006). 16518402 10.1038/ng1752 PMC1435790

[b3] Spitz F. & Furlong E. E. Transcription factors: from enhancer binding to developmental control. Nat. Rev. Genet. 13, 613–626 (2012). 22868264 10.1038/nrg3207

[b4] Muratani M. . Nanoscale chromatin profiling of gastric adenocarcinoma reveals cancer-associated cryptic promoters and somatically acquired regulatory elements. Nat. Commun. 5, 4361 (2014). 25008978 10.1038/ncomms5361

[b5] Bernstein B. E. . Genomic maps and comparative analysis of histone modifications in human and mouse. Cell 120, 169–181 (2005). 15680324 10.1016/j.cell.2005.01.001

[b6] Heintzman N. D. . Distinct and predictive chromatin signatures of transcriptional promoters and enhancers in the human genome. Nat. Genet. 39, 311–318 (2007). 17277777 10.1038/ng1966

[b7] Akhtar-Zaidi B. . Epigenomic enhancer profiling defines a signature of colon cancer. Science 336, 736–739 (2012). 22499810 10.1126/science.1217277 PMC3711120

[b8] Zhang J. A., Mortazavi A., Williams B. A., Wold B. J. & Rothenberg E. V. Dynamic transformations of genome-wide epigenetic marking and transcriptional control establish T cell identity. Cell 149, 467–482 (2012). 22500808 10.1016/j.cell.2012.01.056 PMC3336965

[b9] Thurman R. E. . The accessible chromatin landscape of the human genome. Nature 489, 75–82 (2012). 22955617 10.1038/nature11232 PMC3721348

[b10] Jin F. . A high-resolution map of the three-dimensional chromatin interactome in human cells. Nature 503, 290–294 (2013). 24141950 10.1038/nature12644 PMC3838900

[b11] Hnisz D. . Super-enhancers in the control of cell identity and disease. Cell 155, 934–947 (2013). 24119843 10.1016/j.cell.2013.09.053 PMC3841062

[b12] Loven J. . Selective inhibition of tumor oncogenes by disruption of super-enhancers. Cell 153, 320–334 (2013). 23582323 10.1016/j.cell.2013.03.036 PMC3760967

[b13] Forrester W. C. . A deletion of the human beta-globin locus activation region causes a major alteration in chromatin structure and replication across the entire beta-globin locus. Genes Dev. 4, 1637–1649 (1990). 2249769 10.1101/gad.4.10.1637

[b14] Hovestadt V. . Decoding the regulatory landscape of medulloblastoma using DNA methylation sequencing. Nature 510, 537–541 (2014). 24847876 10.1038/nature13268

[b15] Koch F. . Transcription initiation platforms and GTF recruitment at tissue-specific enhancers and promoters. Nat. Struct. Mol. Biol. 18, 956–963 (2011). 21765417 10.1038/nsmb.2085

[b16] Parker S. C. . Chromatin stretch enhancer states drive cell-specific gene regulation and harbor human disease risk variants. Proc. Natl Acad. Sci. USA 110, 17921–17926 (2013). 24127591 10.1073/pnas.1317023110 PMC3816444

[b17] Chapuy B. . Discovery and characterization of super-enhancer-associated dependencies in diffuse large B cell lymphoma. Cancer Cell 24, 777–790 (2013). 24332044 10.1016/j.ccr.2013.11.003 PMC4018722

[b18] Smiraglia D. J. . Excessive CpG island hypermethylation in cancer cell lines versus primary human malignancies. Hum. Mol. Genet. 10, 1413–1419 (2001). 11440994 10.1093/hmg/10.13.1413

[b19] Ferlay J. . GLOBOCAN 2012 v1.0, Cancer Incidence and Mortality Worldwide: IARC CancerBase No. 11 Vol. 2015 International Agency for Research on Cancer (2012).

[b20] Tan P. & Yeoh K. G. Genetics and molecular pathogenesis of gastric adenocarcinoma. Gastroenterology 149, 1153–1162. e3 (2015). 26073375 10.1053/j.gastro.2015.05.059

[b21] Zouridis H. . Methylation subtypes and large-scale epigenetic alterations in gastric cancer. Sci. Transl. Med. 4, 156ra140 (2012). 10.1126/scitranslmed.3004504 23076357

[b22] Diaz A., Nellore A. & Song J. S. CHANCE: comprehensive software for quality control and validation of ChIP-seq data. Genome Biol. 13, R98 (2012). 23068444 10.1186/gb-2012-13-10-r98 PMC4053734

[b23] Baek S. J. . Integrated epigenomic analyses of enhancer as well as promoter regions in gastric cancer. Oncotarget 7, 25620–25631 (2016). 27016420 10.18632/oncotarget.8239 PMC5041931

[b24] Aran D., Sirota M. & Butte A. J. Systematic pan-cancer analysis of tumour purity. Nat. Commun. 6, 8971 (2015). 26634437 10.1038/ncomms9971 PMC4671203

[b25] Farh K. K. . Genetic and epigenetic fine mapping of causal autoimmune disease variants. Nature 518, 337–343 (2015). 25363779 10.1038/nature13835 PMC4336207

[b26] Li G. . Extensive promoter-centered chromatin interactions provide a topological basis for transcription regulation. Cell 148, 84–98 (2012). 22265404 10.1016/j.cell.2011.12.014 PMC3339270

[b27] Kasowski M. . Extensive variation in chromatin states across humans. Science 342, 750–752 (2013). 24136358 10.1126/science.1242510 PMC4075767

[b28] Roadmap Epigenomics C. . Integrative analysis of 111 reference human epigenomes. Nature 518, 317–330 (2015). 25693563 10.1038/nature14248 PMC4530010

[b29] Wang J. . MALAT1 promotes cell proliferation in gastric cancer by recruiting SF2/ASF. Biomed. Pharmacother. 68, 557–564 (2014). 24857172 10.1016/j.biopha.2014.04.007

[b30] Corradin O. . Combinatorial effects of multiple enhancer variants in linkage disequilibrium dictate levels of gene expression to confer susceptibility to common traits. Genome Res. 24, 1–13 (2014). 24196873 10.1101/gr.164079.113 PMC3875850

[b31] McLean C. Y. . GREAT improves functional interpretation of cis-regulatory regions. Nat. Biotechnol. 28, 495–501 (2010). 20436461 10.1038/nbt.1630 PMC4840234

[b32] Eden E., Navon R., Steinfeld I., Lipson D. & Yakhini Z. GOrilla: a tool for discovery and visualization of enriched GO terms in ranked gene lists. BMC Bioinform. 10, 48 (2009). 10.1186/1471-2105-10-48 PMC2644678 19192299

[b33] Cancer Genome Atlas Research, N. Comprehensive molecular characterization of gastric adenocarcinoma. Nature 513, 202–209 (2014). 25079317 10.1038/nature13480 PMC4170219

[b34] Cristescu R. . Molecular analysis of gastric cancer identifies subtypes associated with distinct clinical outcomes. Nat. Med. 21, 449–456 (2015). 25894828 10.1038/nm.3850

[b35] Tan I. B. . Intrinsic subtypes of gastric cancer, based on gene expression pattern, predict survival and respond differently to chemotherapy. Gastroenterology 141, 476–485 485 e1-11 (2011). 21684283 10.1053/j.gastro.2011.04.042 PMC3152688

[b36] Davies J. O. . Multiplexed analysis of chromosome conformation at vastly improved sensitivity. Nat. Methods 13, 74–80 (2016). 26595209 10.1038/nmeth.3664 PMC4724891

[b37] Thongjuea S., Stadhouders R., Grosveld F. G., Soler E. & Lenhard B. r3Cseq: an R/Bioconductor package for the discovery of long-range genomic interactions from chromosome conformation capture and next-generation sequencing data. Nucleic Acids Res. 41, e132 (2013). 23671339 10.1093/nar/gkt373 PMC3711450

[b38] Kwon M. J. . Claudin-4 overexpression is associated with epigenetic derepression in gastric carcinoma. Lab. Invest. 91, 1652–1667 (2011). 21844869 10.1038/labinvest.2011.117

[b39] Lin X., Yang M., Xia T. & Guo J. Increased expression of long noncoding RNA ABHD11-AS1 in gastric cancer and its clinical significance. Med. Oncol. 31, 42 (2014). 24984296 10.1007/s12032-014-0042-4

[b40] Zhao Z. . Circular chromosome conformation capture (4C) uncovers extensive networks of epigenetically regulated intra- and interchromosomal interactions. Nat. Genet. 38, 1341–1347 (2006). 17033624 10.1038/ng1891

[b41] Zhang B. . A dynamic H3K27ac signature identifies VEGFA-stimulated endothelial enhancers and requires EP300 activity. Genome Res. 23, 917–927 (2013). 23547170 10.1101/gr.149674.112 PMC3668360

[b42] Wang J. L. . Elf3 drives beta-catenin transactivation and associates with poor prognosis in colorectal cancer. Cell Death Dis. 5, e1263 (2014). 24874735 10.1038/cddis.2014.206 PMC4047871

[b43] Lee H. J. . Gene expression profiling of metaplastic lineages identifies CDH17 as a prognostic marker in early stage gastric cancer. Gastroenterology 139, 213–25. e3 (2010). 20398667 10.1053/j.gastro.2010.04.008 PMC2917327

[b44] Yang F. . Long noncoding RNA CCAT1, which could be activated by c-Myc, promotes the progression of gastric carcinoma. J. Cancer Res. Clin. Oncol. 139, 437–445 (2013). 23143645 10.1007/s00432-012-1324-x PMC11824540

[b45] Pasquali L. . Pancreatic islet enhancer clusters enriched in type 2 diabetes risk-associated variants. Nat. Genet. 46, 136–143 (2014). 24413736 10.1038/ng.2870 PMC3935450

[b46] Joseph C. G. . Association of the autoimmune disease scleroderma with an immunologic response to cancer. Science 343, 152–157 (2014). 24310608 10.1126/science.1246886 PMC4038033

[b47] Siersbaek R. . Transcription factor cooperativity in early adipogenic hotspots and super-enhancers. Cell Rep. 7, 1443–1455 (2014). 24857652 10.1016/j.celrep.2014.04.042

[b48] Griffon A. . Integrative analysis of public ChIP-seq experiments reveals a complex multi-cell regulatory landscape. Nucleic Acids Res. 43, e27 (2015). 25477382 10.1093/nar/gku1280 PMC4344487

[b49] Heinz S. . Simple combinations of lineage-determining transcription factors prime cis-regulatory elements required for macrophage and B cell identities. Mol. Cell 38, 576–589 (2010). 20513432 10.1016/j.molcel.2010.05.004 PMC2898526

[b50] Zambelli F., Pesole G. & Pavesi G. PscanChIP: finding over-represented transcription factor-binding site motifs and their correlations in sequences from ChIP-Seq experiments. Nucleic Acids Res. 41, W535–W543 (2013). 23748563 10.1093/nar/gkt448 PMC3692095

[b51] Barros R., Freund J. N., David L. & Almeida R. Gastric intestinal metaplasia revisited: function and regulation of CDX2. Trends Mol. Med. 18, 555–563 (2012). 22871898 10.1016/j.molmed.2012.07.006

[b52] Chia N. Y. . Regulatory crosstalk between lineage-survival oncogenes KLF5, GATA4 and GATA6 cooperatively promotes gastric cancer development. Gut 64, 707–719 (2015). 25053715 10.1136/gutjnl-2013-306596

[b53] Zang Z. J. . Exome sequencing of gastric adenocarcinoma identifies recurrent somatic mutations in cell adhesion and chromatin remodeling genes. Nat. Genet. 44, 570–574 (2012). 22484628 10.1038/ng.2246

[b54] Wang K. . Exome sequencing identifies frequent mutation of ARID1A in molecular subtypes of gastric cancer. Nat. Genet. 43, 1219–1223 (2011). 22037554 10.1038/ng.982

[b55] Asada K. . Demonstration of the usefulness of epigenetic cancer risk prediction by a multicentre prospective cohort study. Gut 64, 388–396 (2015). 25379950 10.1136/gutjnl-2014-307094 PMC4345890

[b56] Orlando D. A. . Quantitative ChIP-Seq normalization reveals global modulation of the epigenome. Cell Rep. 9, 1163–1170 (2014). 25437568 10.1016/j.celrep.2014.10.018

[b57] Zhang X. . Identification of focally amplified lineage-specific super-enhancers in human epithelial cancers. Nat. Genet. 48, 176–182 (2015). 26656844 10.1038/ng.3470 PMC4857881

[b58] Cowper-Sal lari R. . Breast cancer risk-associated SNPs modulate the affinity of chromatin for FOXA1 and alter gene expression. Nat. Genet. 44, 1191–1198 (2012). 23001124 10.1038/ng.2416 PMC3483423

[b59] Zanke B. W. . Genome-wide association scan identifies a colorectal cancer susceptibility locus on chromosome 8q24. Nat. Genet. 39, 989–994 (2007). 17618283 10.1038/ng2089

[b60] Shen L. . Clinical significance of POU5F1P1 rs10505477 polymorphism in Chinese gastric cancer patients receving cisplatin-based chemotherapy after surgical resection. Int. J. Mol. Sci. 15, 12764–12777 (2014). 25046748 10.3390/ijms150712764 PMC4139873

[b61] Garrison W. D. . Hepatocyte nuclear factor 4alpha is essential for embryonic development of the mouse colon. Gastroenterology 130, 1207–1220 (2006). 16618389 10.1053/j.gastro.2006.01.003 PMC3581272

[b62] Silberg D. G., Swain G. P., Suh E. R. & Traber P. G. Cdx1 and cdx2 expression during intestinal development. Gastroenterology 119, 961–971 (2000). 11040183 10.1053/gast.2000.18142

[b63] Ongen H. . Putative cis-regulatory drivers in colorectal cancer. Nature 512, 87–90 (2014). 25079323 10.1038/nature13602

[b64] Dixon J. R. . Topological domains in mammalian genomes identified by analysis of chromatin interactions. Nature 485, 376–380 (2012). 22495300 10.1038/nature11082 PMC3356448

[b65] Silberg D. G. . Cdx2 ectopic expression induces gastric intestinal metaplasia in transgenic mice. Gastroenterology 122, 689–696 (2002). 11875002 10.1053/gast.2002.31902

[b66] Verzi M. P., Shin H., San Roman A. K., Liu X. S. & Shivdasani R. A. Intestinal master transcription factor CDX2 controls chromatin access for partner transcription factor binding. Mol. Cell Biol. 33, 281–292 (2013). 23129810 10.1128/MCB.01185-12 PMC3554120

[b67] Mansour M. R. . Oncogene regulation. An oncogenic super-enhancer formed through somatic mutation of a noncoding intergenic element. Science 346, 1373–1377 (2014). 25394790 10.1126/science.1259037 PMC4720521

[b68] Das K. . Mutually exclusive FGFR2, HER2, and KRAS gene amplifications in gastric cancer revealed by multicolour FISH. Cancer Lett. 353, 167–175 (2014). 25086186 10.1016/j.canlet.2014.07.021

[b69] Mizukami T. . EGFR and HER2 signals play a salvage role in MEK1-mutated gastric cancer after MEK inhibition. Int. J. Oncol. 47, 499–505 (2015). 26081723 10.3892/ijo.2015.3050

[b70] Grygielewicz P. . Epithelial-mesenchymal transition confers resistance to selective FGFR inhibitors in SNU-16 gastric cancer cells. Gastric Cancer 19, 53–62 (2016). 25407459 10.1007/s10120-014-0444-1 PMC4688307

[b71] McVicker G. . Identification of genetic variants that affect histone modifications in human cells. Science 342, 747–749 (2013). 24136359 10.1126/science.1242429 PMC3947669

[b72] Li H. & Durbin R. Fast and accurate long-read alignment with Burrows-Wheeler transform. Bioinformatics 26, 589–595 (2010). 20080505 10.1093/bioinformatics/btp698 PMC2828108

[b73] Ruffalo M., Koyuturk M., Ray S. & LaFramboise T. Accurate estimation of short read mapping quality for next-generation genome sequencing. Bioinformatics 28, i349–i355 (2012). 22962451 10.1093/bioinformatics/bts408 PMC3436835

[b74] Xie W. . Epigenomic analysis of multilineage differentiation of human embryonic stem cells. Cell 153, 1134–1148 (2013). 23664764 10.1016/j.cell.2013.04.022 PMC3786220

[b75] Hon G., Ren B. & Wang W. ChromaSig: a probabilistic approach to finding common chromatin signatures in the human genome. PLoS Comput. Biol. 4, e1000201 (2008). 18927605 10.1371/journal.pcbi.1000201 PMC2556089

[b76] Davoli T. . Cumulative haploinsufficiency and triplosensitivity drive aneuploidy patterns and shape the cancer genome. Cell 155, 948–962 (2013). 24183448 10.1016/j.cell.2013.10.011 PMC3891052

[b77] Splinter E., de Wit E., van de Werken H. J., Klous P. & de Laat W. Determining long-range chromatin interactions for selected genomic sites using 4C-seq technology: from fixation to computation. Methods 58, 221–230 (2012). 22609568 10.1016/j.ymeth.2012.04.009

[b78] Ramlee M. K., Yan T., Cheung A. M., Chuah C. T. & Li S. High-throughput genotyping of CRISPR/Cas9-mediated mutants using fluorescent PCR-capillary gel electrophoresis. Sci. Rep. 5, 15587 (2015). 26498861 10.1038/srep15587 PMC4620477

[b79] Qin R., Wang N. N., Chu J. & Wang X. Expression and significance of homeodomain protein Cdx2 in gastric carcinoma and precancerous lesions. World J. Gastroenterol. 18, 3296–3302 (2012). 22783055 10.3748/wjg.v18.i25.3296 PMC3391768

