# Supplementary material for: Epigenomic profiling of primary gastric adenocarcinoma reveals super-enhancer heterogeneity
Source: Nat Commun. 2016 Sep 28;7:12983. doi: 10.1038/ncomms12983 (PMC5052795; doi:10.1038/ncomms12983)
Supplement: Supplementary Information — Supplementary Figures 1-17, Supplementary Tables 1-6, Supplementary Discussion and Supplementary References [file ncomms12983-s1.pdf]

Supplementary Figures

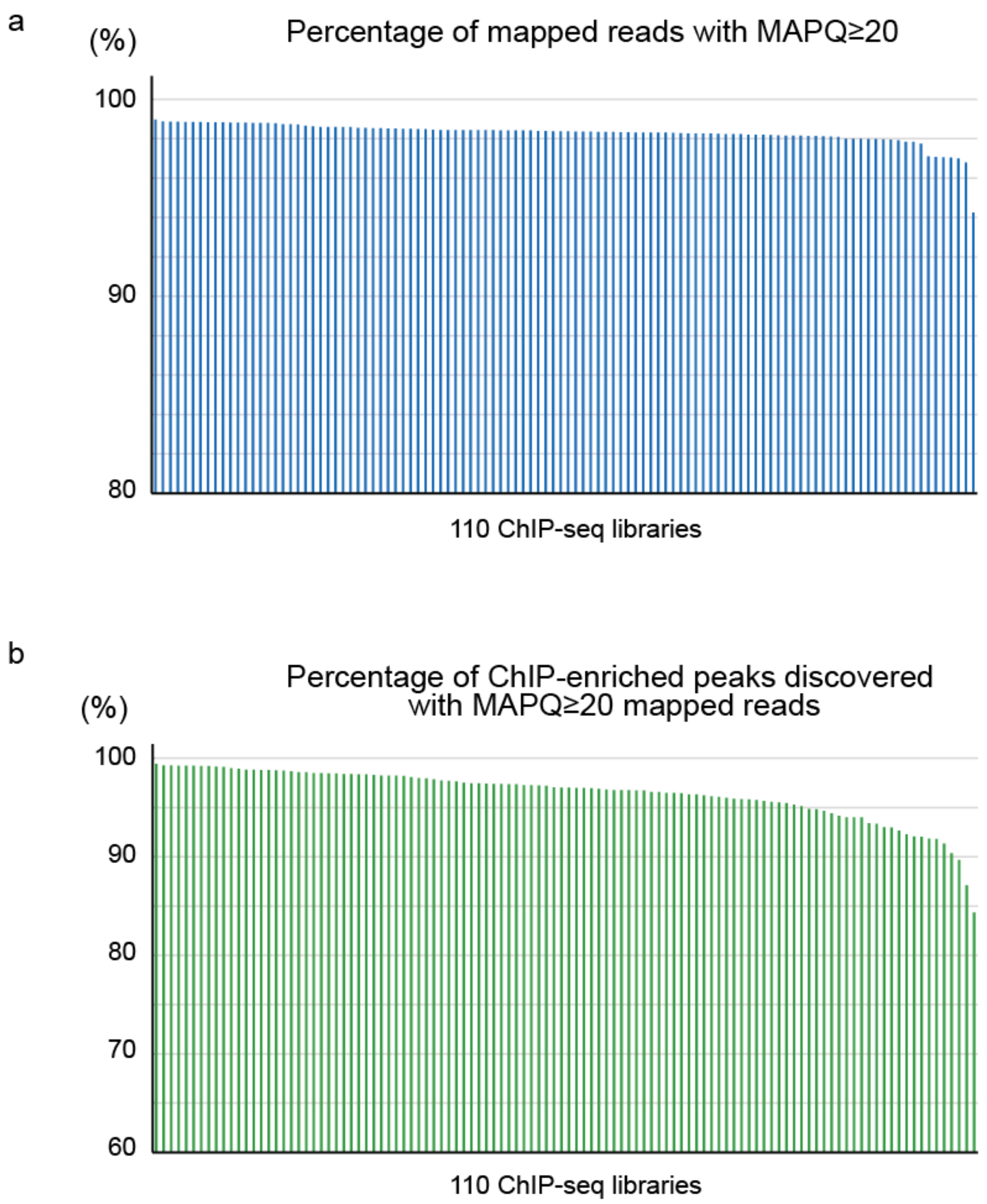

Supplementary Figure 1: Comparisons between different mapping quality filters (MAPQ≥10 and MAPQ≥20).
a. Percentage of mapped reads detected using MAPQ≥20 compared to the total mapped reads using MAPQ≥10.
b. Percentage of ChIP-enriched peaks discovered using MAPQ≥20 compared to the total number of ChIP-enriched peaks using MAPQ≥10.

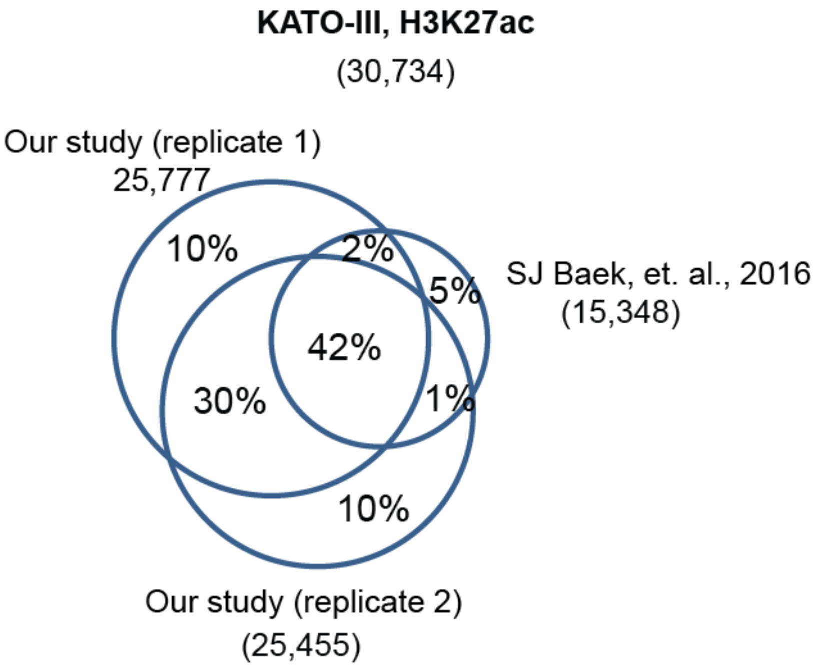

Supplementary Figure 2: Concordance of H3K27ac-enriched peaks among biological replicates from KATO-III cells. Replicate 1 and 2 were generated using Nano-ChIPseq, while data from Baek et al. (2016) was created using conventional ChIPseq methods. The total number of mapped reads from replicate 1 and 2 is >10x more than the Baek et al. data[1], and therefore more peaks were detected in our replicates. Peaks from replicates were merged using BEDTools. Using this approach, we identified 30,734 unique peaks. Percentage of overlapping peaks found in replicates compared to the total number unique peaks was computed.

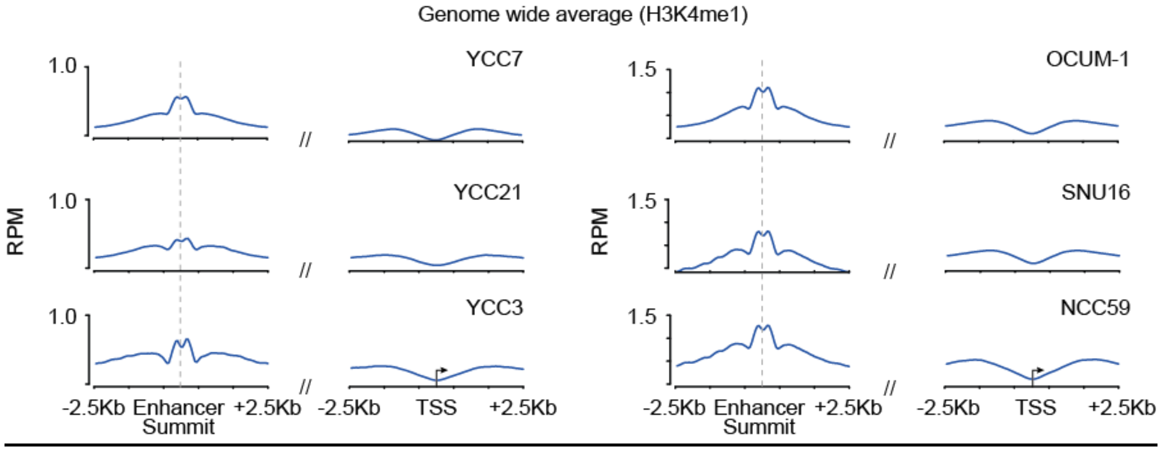

Supplementary Figure 3: Genome-wide H3K4me1 signals flanking distal predicted enhancers and active TSSs in gastric cancer cell lines.

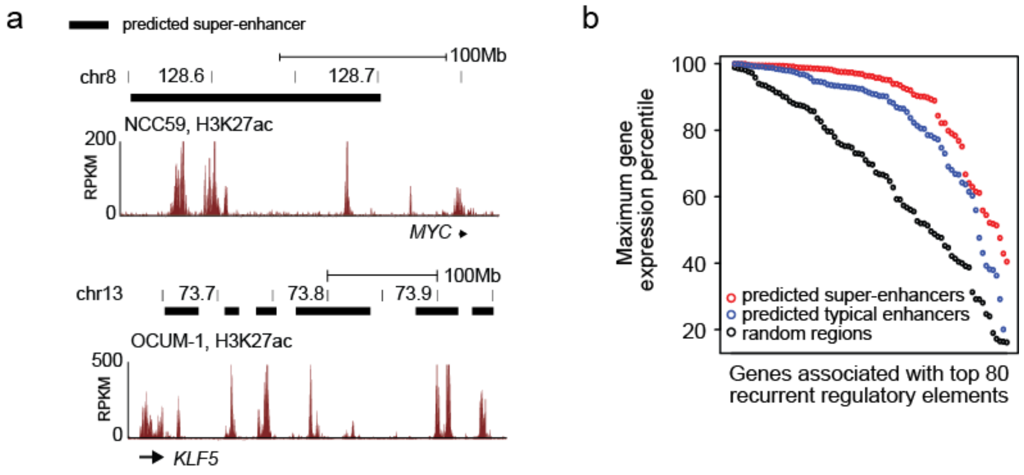

Supplementary Figure 4: Predicted super-enhancers in GC cell lines.
a. *KLF5*- and *MYC*-associated predicted super-enhancers in OCUM-1 and NCC59, respectively.
b. Expression levels of genes (in percentile units, across the cell lines) linked to the top recurrent predicted super-enhancers (red) and predicted typical enhancers (blue). An identical number of randomly chosen genes (black) was used as the reference. Genes were sorted by percentiles in the order from highest to smallest.

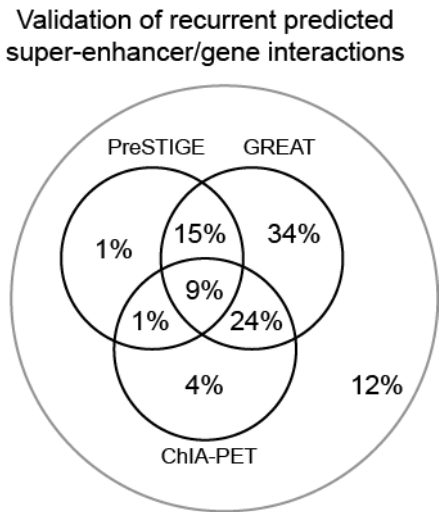

Supplementary Figure 5: Validation of recurrent predicted super-enhancer/gene interactions using public data sets. Percentage values reflect the original predicted super-enhancer/gene assignments (see Results and Methods).

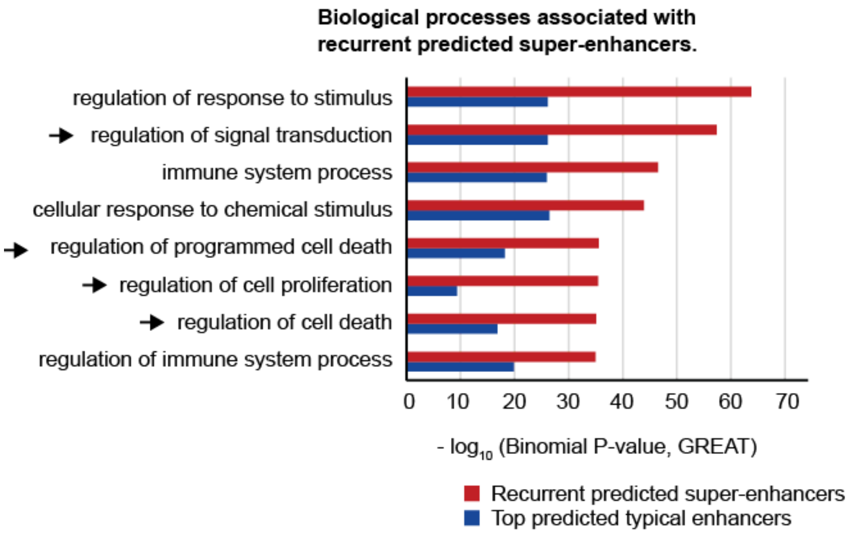

Supplementary Figure 6: Biological processes associated with recurrent predicted super-enhancers using GREAT analysis tool. Processes highlighted by black arrows refer to processes observed by both GOrilla (see Results) and GREAT.

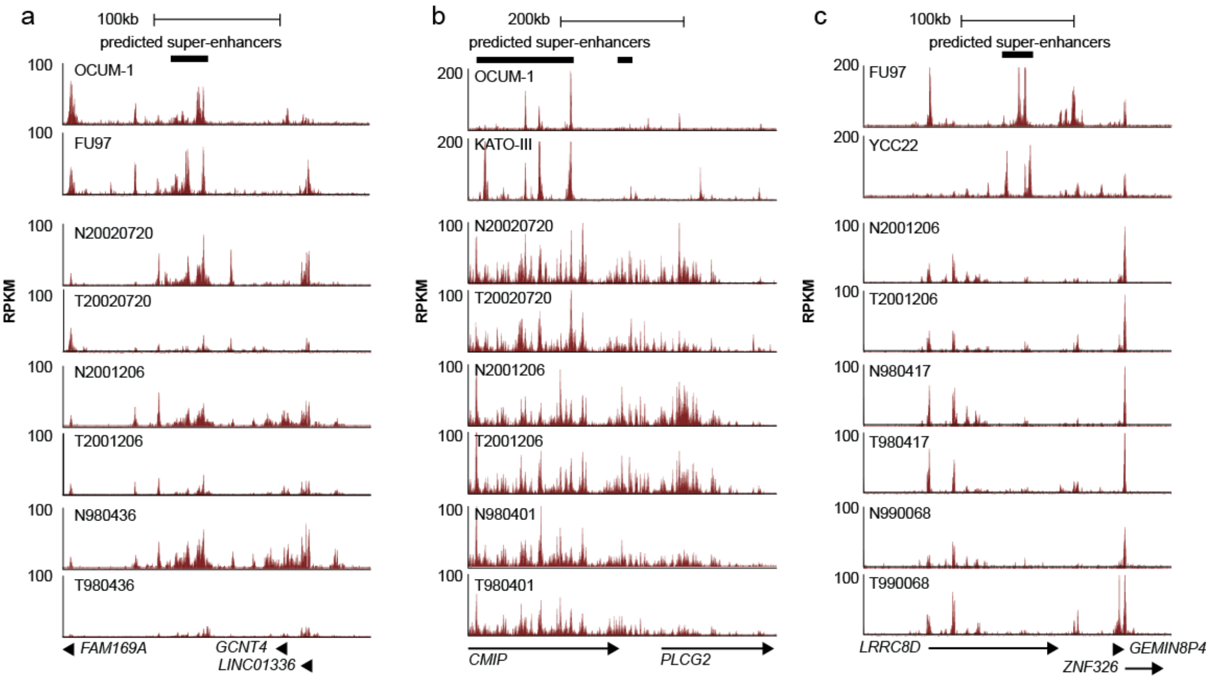

Supplementary Figure 7: Categorization of cell-line derived predicted super-enhancers using histone H3K27ac profiles from primary samples.
a. A somatic loss predicted super-enhancer in three tumor(T)/matched normal(N) pairs at the *GCNT4* locus.
b. An unaltered predicted super-enhancer in T/N20020720, T/N2001206 and T/N980401 at the *CMIP* locus.
c. A predicted super-enhancer detected in FU97 and YCC22 GC cells shows an inactive state in three T/N pairs at the *ZNF326* locus.

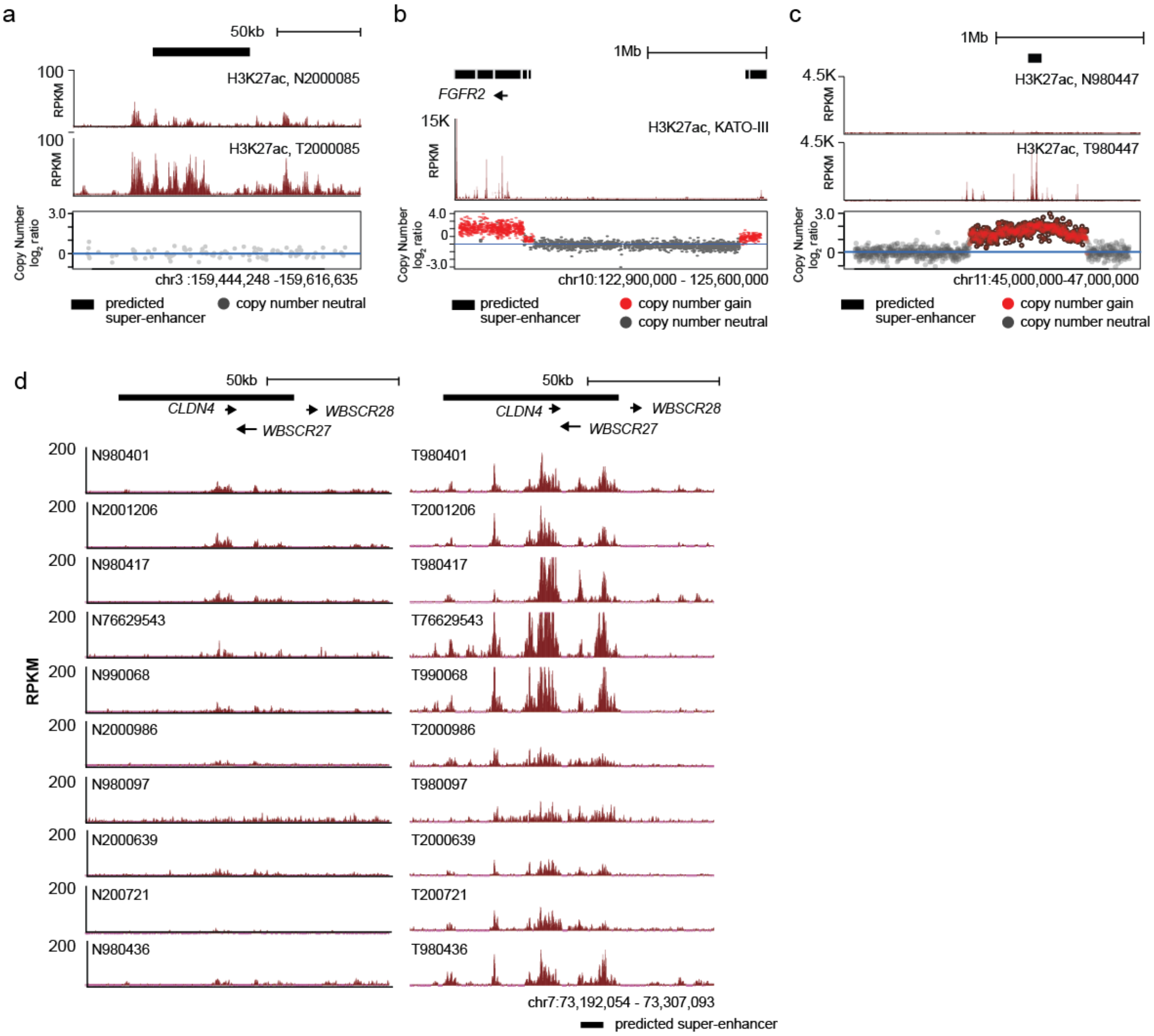

Supplementary Figure 8: Association between copy number alterations and predicted super-enhancers.

a. An example of a somatic gain predicted super-enhancer detected in a copy number neutral region.

b. *FGFR2*-associated predicted super-enhancers detected at regions of somatic copy number gain in KATO-III cells.

c. A somatic gain predicted super-enhancer detected in a region with copy number gain in T/N980447.

d. A highly recurrent somatic gain (H3K27ac) predicted super-enhancer was detected at the *CLDN4* locus. This region was not associated with copy number gain.

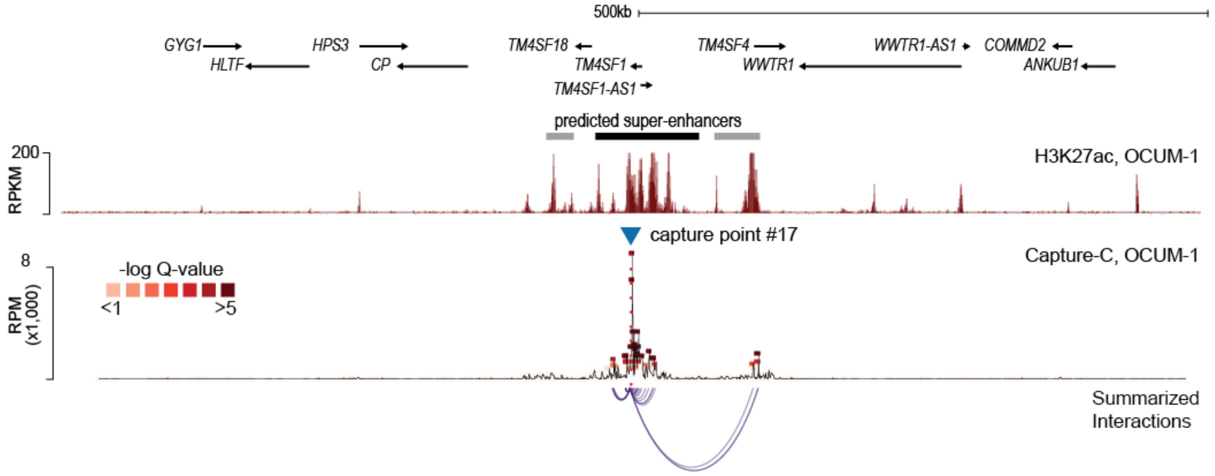

Supplementary Figure 9: Long-range interactions between a predicted super-enhancer (black rectangle) at *TM4SF1* locus and the *TM4SF4* promoter detected in OCUM-1 cells using Capture-C technology. The bottom track indicates the summarized interactions from the capture point #17.

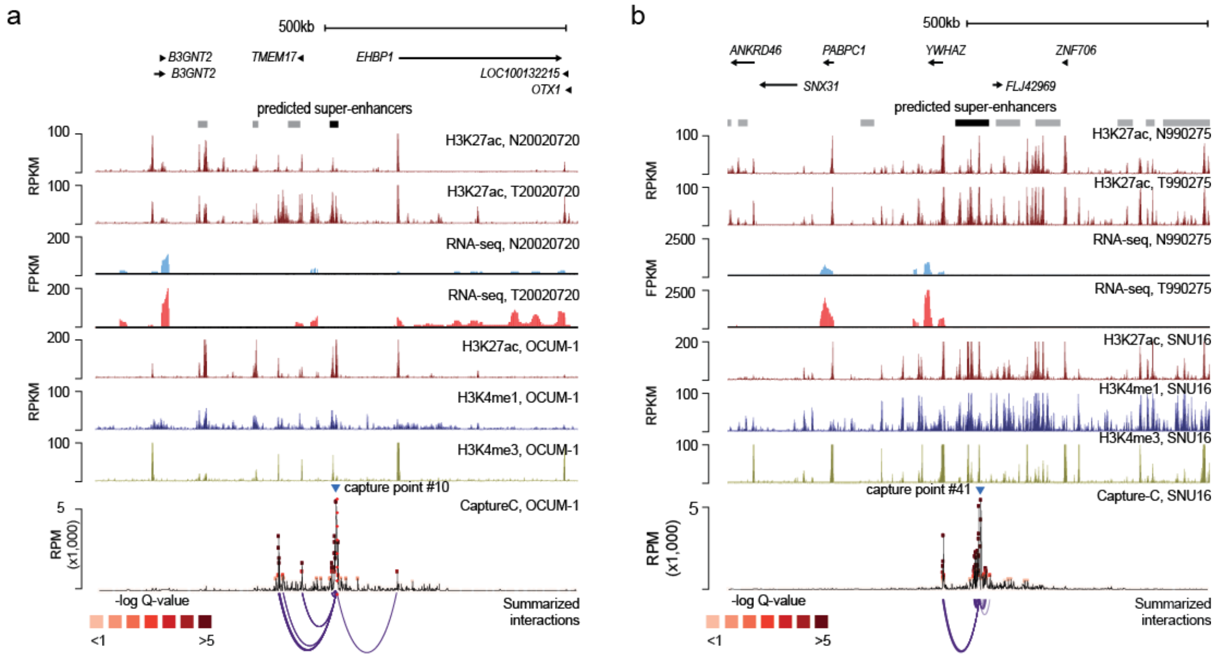

Supplementary Figure 10: Capture-C interaction profiles.

a. Interactions from the *EHBP1* predicted super-enhancer (black rectangle) to promoters of *TMEM1* and *EHBP1* genes. The predicted super-enhancer was detected in OCUM-1 cells, showed somatic gain in primary tumor T20020720 and is associated with up-regulated expression of *TMEM1* and *EBHP1*.

b. Interactions from a predicted super-enhancer (black rectangle) at the *YWHAZ* locus to the promoter of *YWHAZ*. The predicted super-enhancer was detected in SNU16 cells, showed somatic gain in the primary tumor sample T990275 and is associated with up-regulated expression of *YWHAZ*.

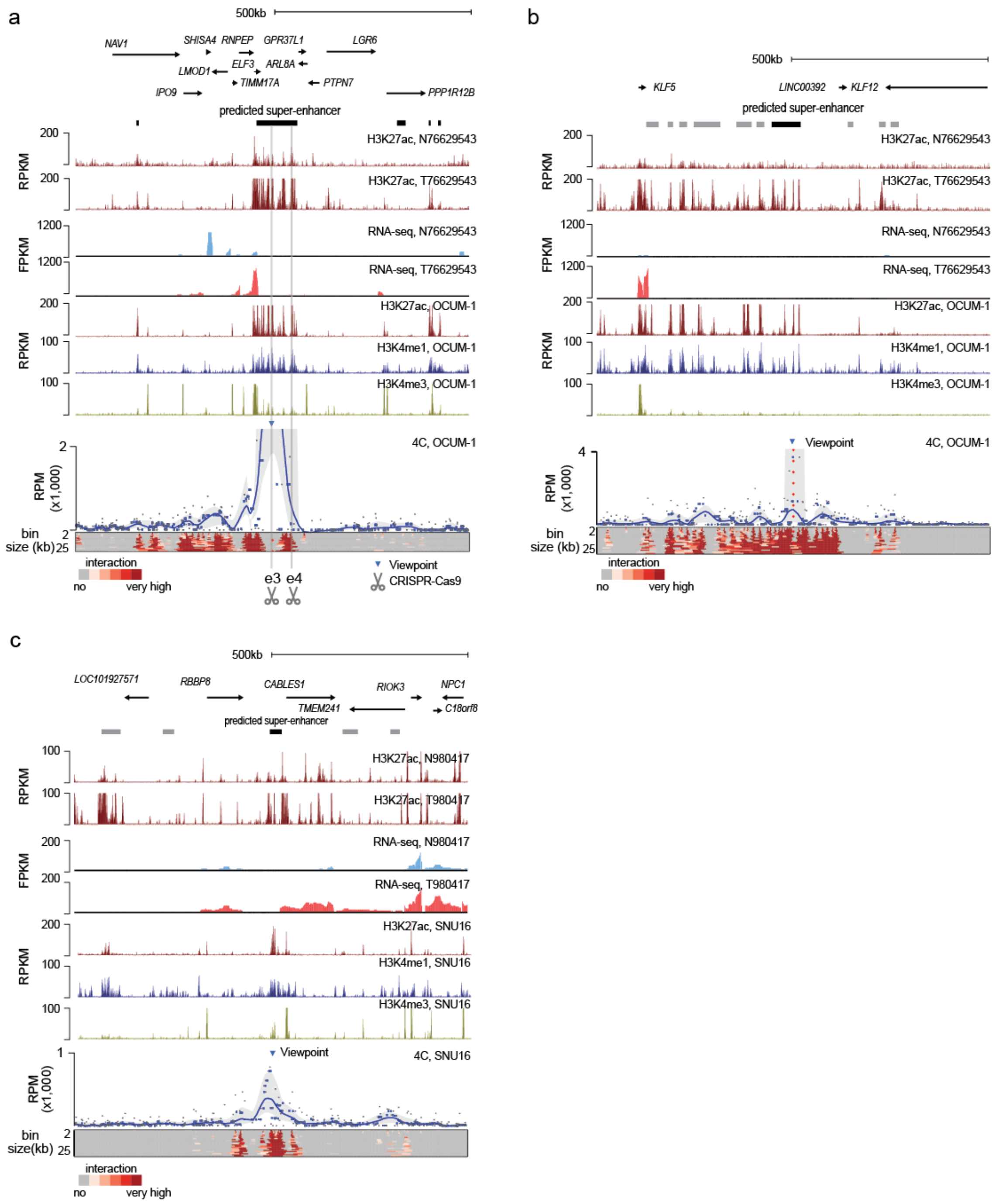

Supplementary Figure 11: 4C interaction profiles.

a. Example of a somatic gain predicted super-enhancer at the *ELF3* locus and interactions with neighbouring genes, such as *ELF3*, *RNPEP*, *ARL8A* and *LMOD1*. Somatic gain activity is associated with up-regulation of *ELF3* in primary GCs. Interactions (Q<0.05, r3Cseq) were detected in OCUM-1 cells using 4C. The 4C signal plot (in units of RPM) was generated using the Basic4CSeq package. Two constituent enhancers, e3 and e4 were deleted independently in OCUM-1 cells using CRISPR/Cas9 genome editing technology.

b. Long-range interactions between a predicted super-enhancer at *KLF5* locus and the *KLF5* promoter were detected in OCUM-1 cells. Somatic gain activity in the primary tumor (T76629543) is associated with up-regulation of *KLF5* expression in the matched sample.

c. Interactions of a predicted super-enhancer at the *CABLES1* locus to neighbouring non-coding regions and promoters of genes, including *CABLES1* and *RIOK3*.

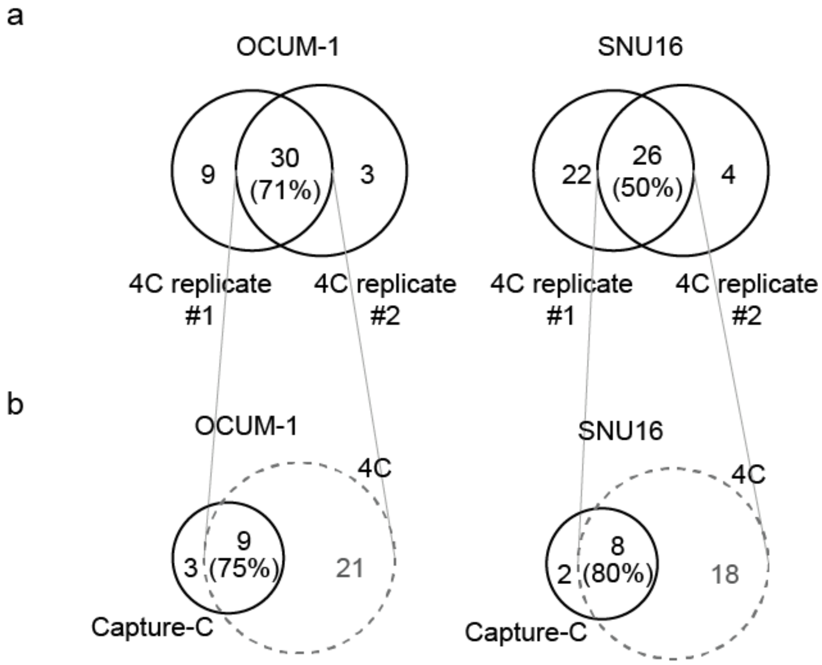

Supplementary Figure 12: Comparing interaction profiles from Capture-C and 4C.
a. Venn diagrams show the overlap of predicted super-enhancer/gene interactions (from 4C) between two biological replicates from OCUM-1 and SNU16 cells. The concordance between replicates was computed (percentage in brackets) with respect to all identified interactions.
b. Venn diagrams show the overlap of predicted super-enhancer/gene interactions (from Capture-C) with the concordant set of interactions from 4C in the same cells. 75%-80% of the interactions identified by using Capture-C were rediscovered in the results using 4C.

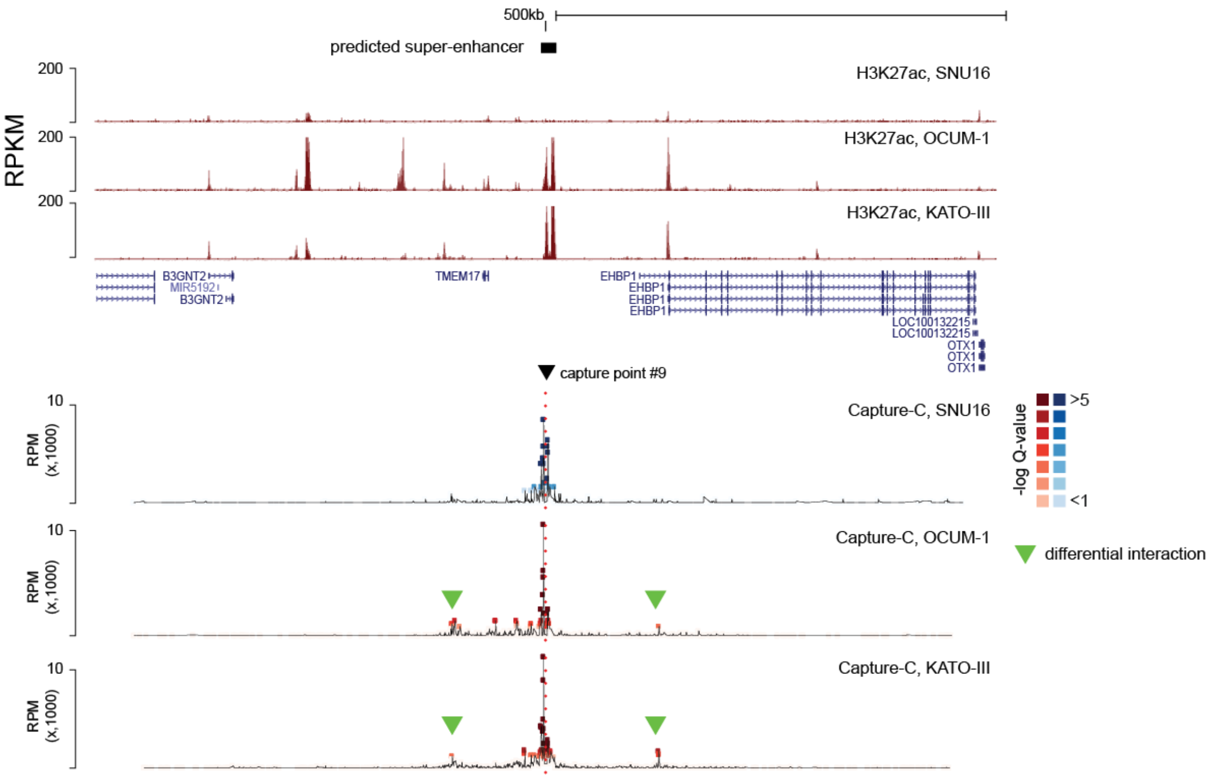

Supplementary Figure 13: An example of correlation between predicted super-enhancer activity and the presence of long-range interactions. Long-range interactions (green triangle) to the *EHBP1* promoter were detected with a predicted super-enhancer (black rectangle) active in OCUM-1 and KATO-III cells. Such interactions were not observed in SNU16 cells where the predicted super-enhancer was also not detected.

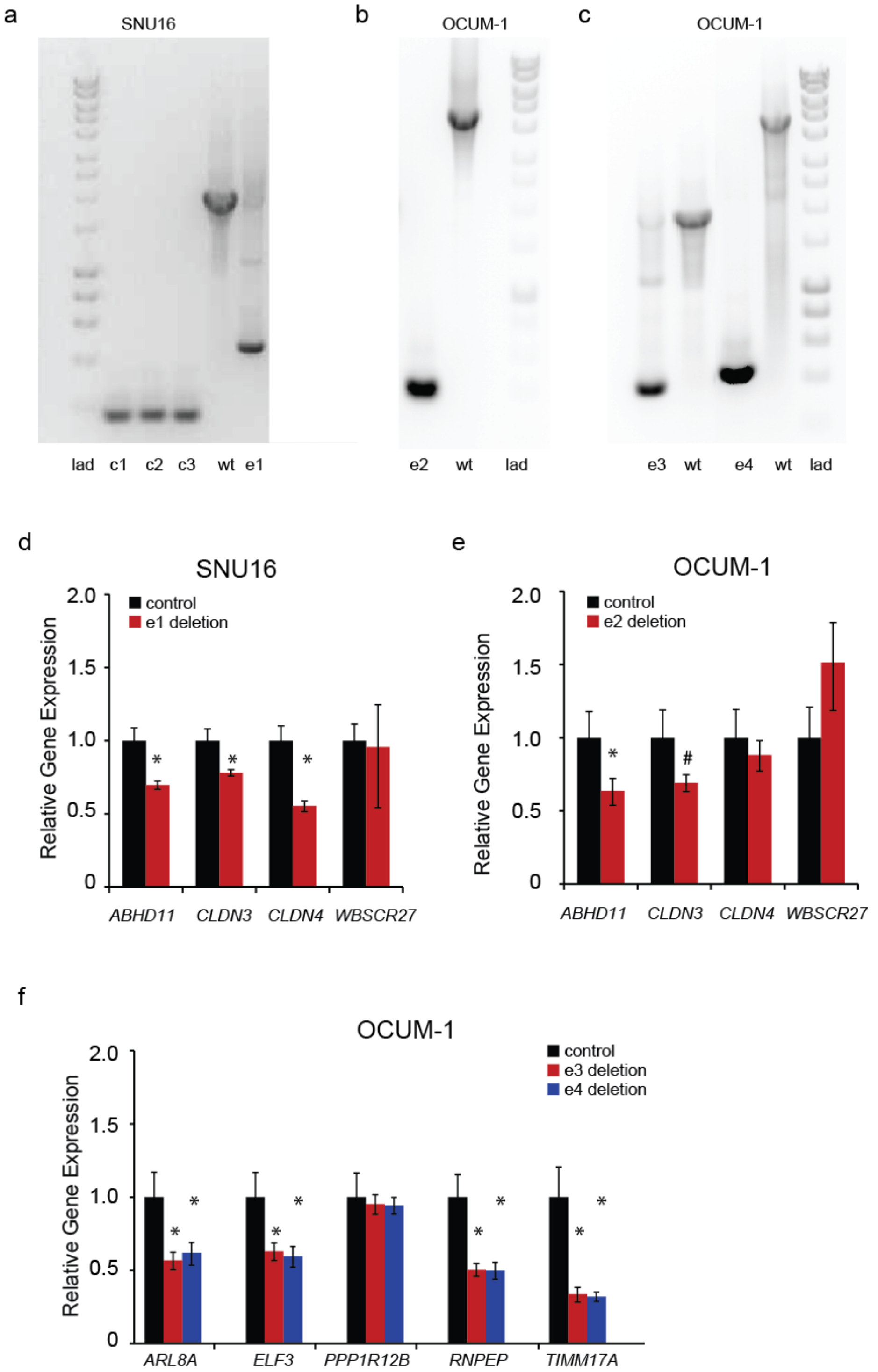

Supplementary Figure 14: Predicted enhancer deletion using CRISPR/Cas9 deletion. PCR analysis of CRISPR/Cas9 deletion of a) the constituent enhancer, e1 in SNU16, b) the constituent enhancer, e2 in OCUM-1, c) the constituent enhancers, e3 and e4 in OCUM-1. (e-f) Differential gene expression between mutant (with one predicted enhancer deletion) and wild type cells was performed using RT-qPCR in OCUM-1 and SNU16 cells. Pooled cells were analysed. *P<0.05, # P = 0.055, one-sided t-test; wt: wild type; lad: DNA ladder (Bioline HyperLadder I); c1-c3: wild type cells using GAPDH primers.

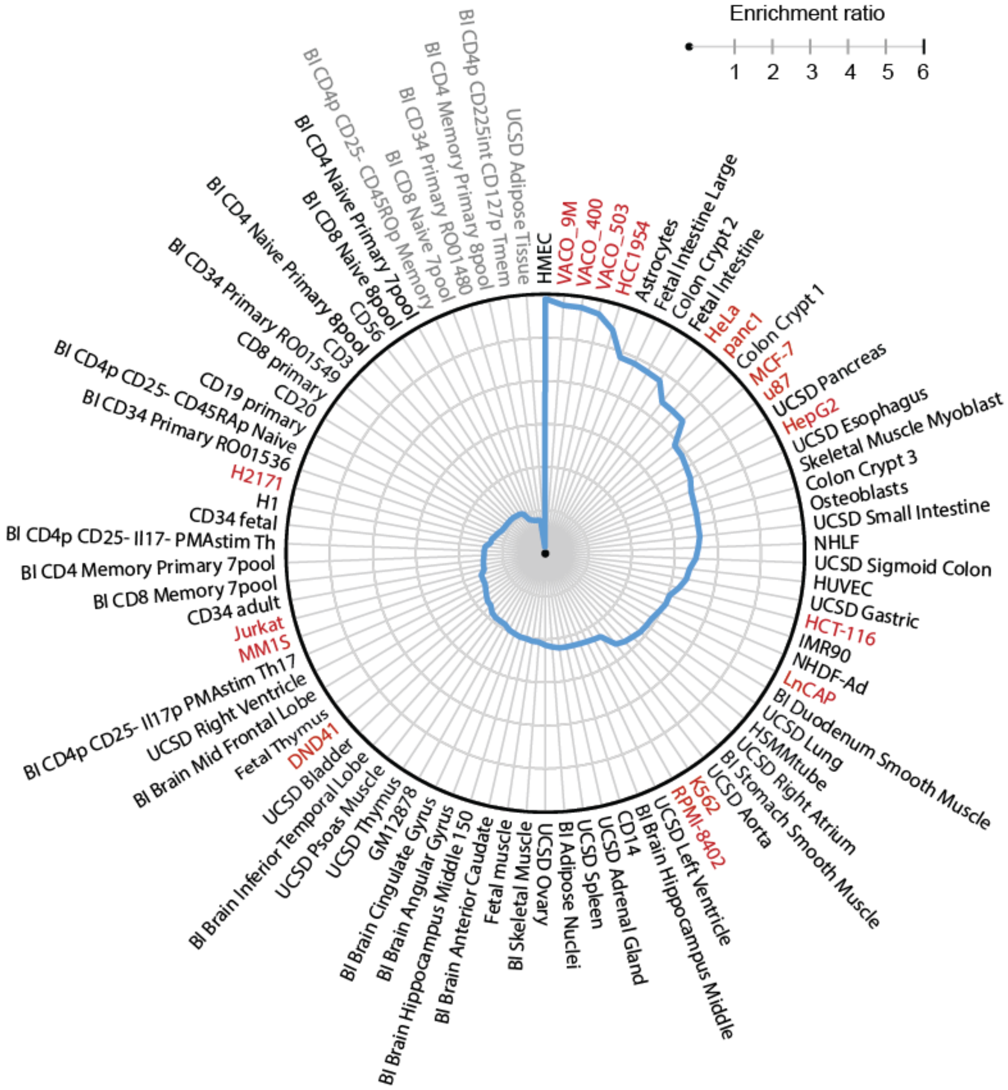

Supplementary Figure 15: Landscape of GC-associated predicted super-enhancers in other cell and tissue types.

Enrichment ratios of recurrent somatic gain predicted super-enhancers identified in GC overlapping with super-enhancers detected in 86 cell and tissue samples compared to randomly selected regions. Cancer cell lines are labelled in red; Samples with statistically insignificant ($P > 0.001$) enrichment ratios are in grey.

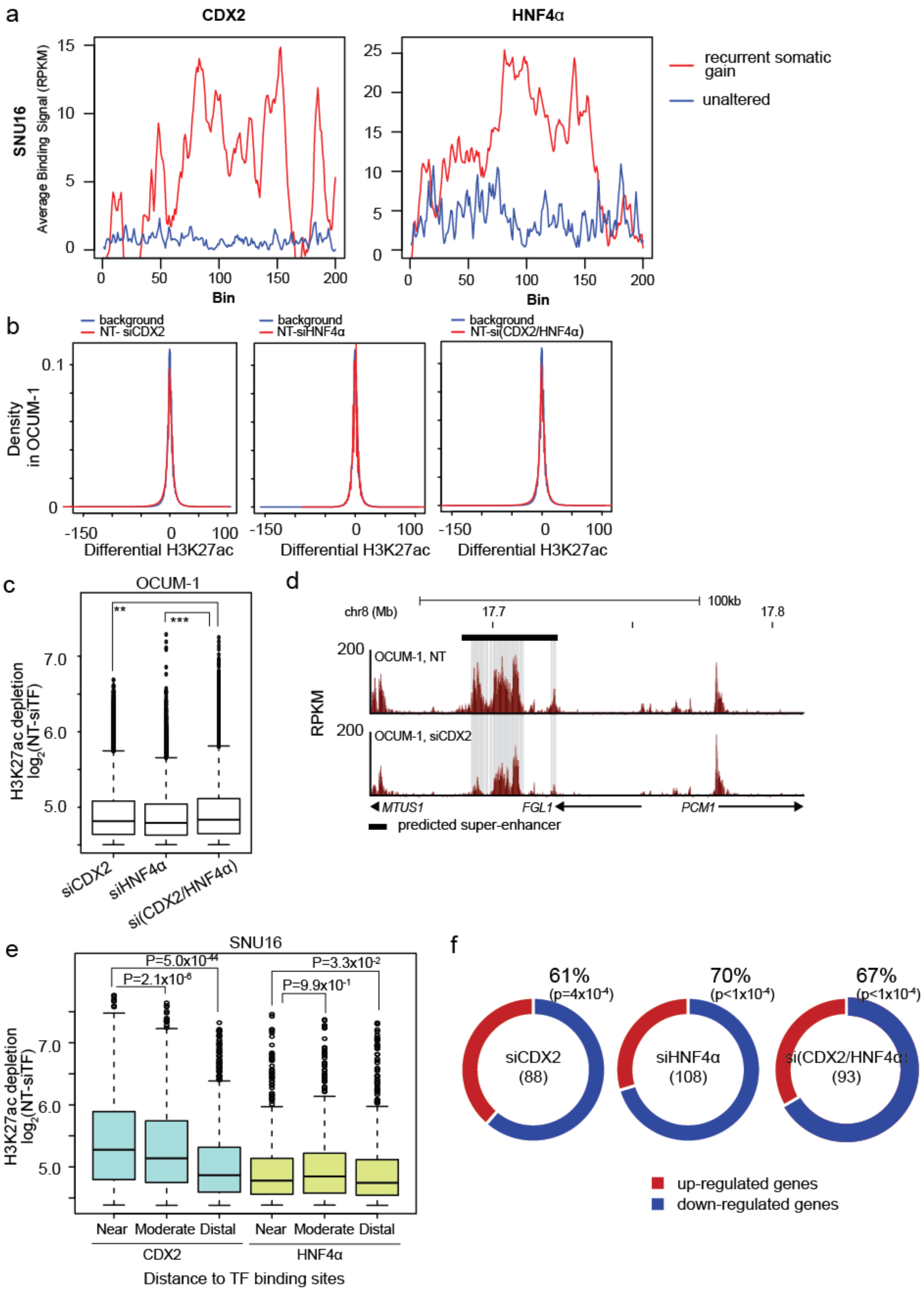
a
CDX2
HNF4α
SNU16
Average Binding Signal (RPKM)
Bin
recurrent somatic gain
unaltered
b
background
NT- siCDX2
NT-siHNF4α
NT-si(CDX2/HNF4α)
Density in OCUM-1
Differential H3K27ac
c
OCUM-1
H3K27ac depletion log2(NT-siTF)
siCDX2
siHNF4α
si(CDX2/HNF4α)
d
chr8 (Mb)
100kb
17.7
17.8
RPKM
OCUM-1, NT
OCUM-1, siCDX2
MTUS1
FGL1
PCM1
predicted super-enhancer
e
SNU16
P=5.0x10-44
P=2.1x10-6
P=3.3x10-2
P=9.9x10-1
Near
Moderate
Distal
CDX2
HNF4α
Distance to TF binding sites
f
61%
(p=4x10-4)
70%
(p<1x10-4)
67%
(p<1x10-4)
siCDX2 (88)
siHNF4α (108)
si(CDX2/HNF4α) (93)
up-regulated genes
down-regulated genes

Supplementary Figure 16: Consequences of transcription factor-silencing on histone modifications and gene expression.

a. Differential CDX2 (left) and HNF4α (right) average binding signal analysis between recurrent somatic gain predicted super-enhancers and unaltered predicted super-enhancers. The predicted super-enhancers were also active in SNU16.

b. Global changes in H3K27ac after silencing one or two transcription factors simultaneously (red). Background changes are created from the difference between two controls (NT_{CDX2} and $NT_{HNF4\alpha}$).

c. Magnitude of H3K27ac depletion after silencing of transcription factor(s) in OCUM-1 cells.

d. Visual example showing H3K27ac depletion in a predicted super-enhancer at the *FGL1* locus after *CDX2* silencing in OCUM-1 cells.

e. Association between H3K27ac depletion in somatic gain predicted super-enhancers relative to CDX2 or HNF4α binding sites in SNU16 cells. Distances were uniformly distributed classified into three categories: near, moderate and distal to the binding sites. Statistical significance was evaluated using a one-sided Wilcoxon rank sum test.

f. Gene expression associated with somatic gain predicted super-enhancers in OCUM-1 was examined after the silencing of single or double transcription factors simultaneously (NT-siTF). The percentage of genes showing changes in expression (FPKM difference > 0 as down-regulation; < 0 as up-reglation) is indicated. The proportion of down-regulated genes was tested using an empirical approach (see Methods).

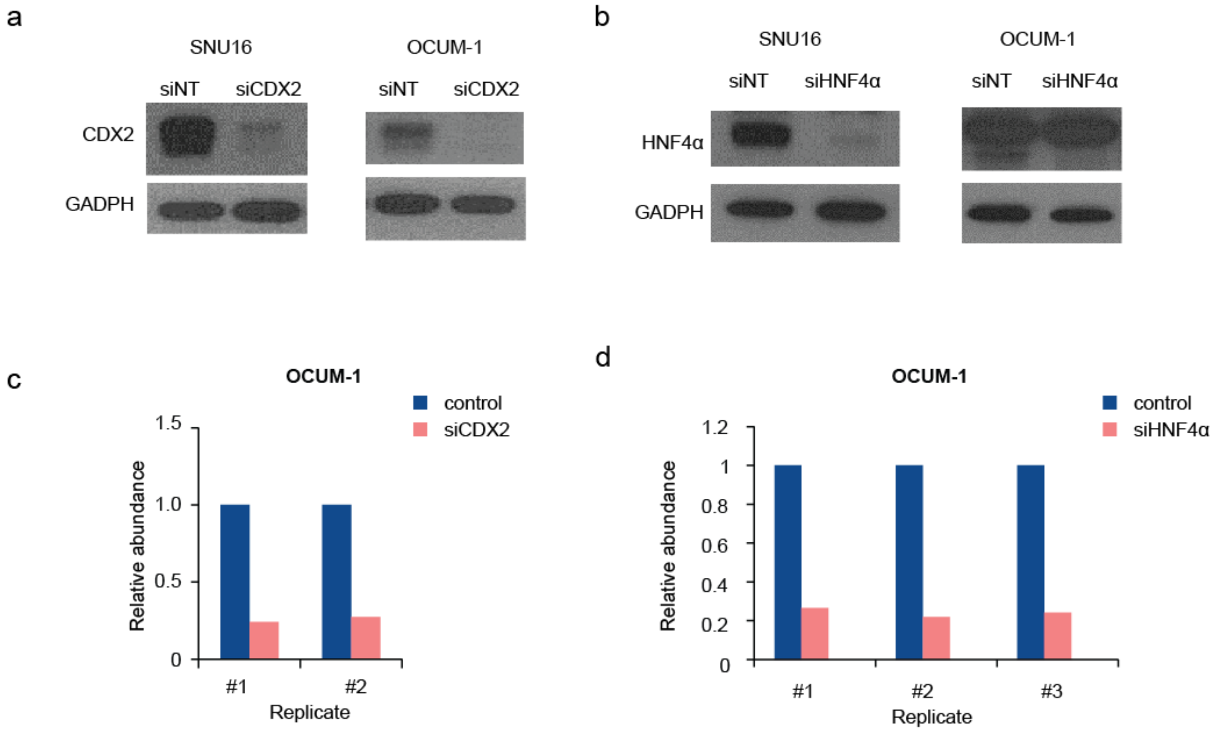

Supplementary Figure 17: CDX2, HNF4α knockdown efficiency by Western blotting and real time (RT) PCR.
a. Western blot measuring CDX2 protein abundance before (siNT) and after CDX2 knockdown (siCDX2) in SNU16 and OCUM-1 cells. GADPH protein abundance was used as a control.
b. Western blot measuring HNF4α protein abundance before (siNT) and after HNF4α knockdown (siHNF4α) in SNU16 and OCUM-1 cells. GADPH protein abundance was used as a control.
c. Relative RNA abundance of *CDX2* to control was measured using RT-PCR in two replicates in OCUM-1 cells.
d. Relative RNA abundance of *HNF4α* to control was measured using RT-PCR in three replicates in OCUM-1 cells.

Supplementary Tables

Supplementary Table 1: Clinical information of patients used in histone ChIP-seq, RNA-seq, Affymetrix SNP6.0 arrays and Infinium HumanMethylation 450K BeadChip arrays.

Patient ID	Tumor content	Molecular Subtype	Lauren's classification	AJCC7
**2000085**	95%	GS	intestinal	1B
**2000639**	60%	GS	intestinal	4
**2000721**	70%	GS	diffuse	4
**2000877**	>90%	CIN	intestinal	2A
**2000986**	80%	GS	diffuse	4
**2001206**	90%	CIN	diffuse	4
**20020720**	80%	CIN	intestinal	2A
**20021007**	85%	GS	intestinal	2A
**76629543**	80%	CIN	intestinal	3A
**980097**	70%	EBV	mixed/OTHERS	2A
**980319**	70%	GS	mixed/OTHERS	3A
**980401**	90%	GS	diffuse	3A
**980417**	80%	GS	diffuse	3C
**980436**	80%	GS	intestinal	3A
**980437**	90%	CIN	intestinal	3C
**980447**	40%	CIN	intestinal	4
**990068**	95%	GS	diffuse	3B
**990275**	50%	CIN	intestinal	2B
**990489**	90%	CIN	mixed/OTHERS	1B

Supplementary Table 2: Gastric cancer cell line information

No.	Cell-line	Histological sub-type of GC	Derivation	Reference
1	FU97	Diffuse adenocarcinoma;poorly differentiated; Lymph node metastasis and pancreatic metastasis were observed	primary stomach	[2]
2	KATOIII	SRCC	pleural effusion	[3]
3	MKN7	Well-differentiated tubular adenocarcinoma	lymph node	[4]
4	NCC-59	Moderately differentiated tubular adenocarcinoma	primary stomach	[5]
5	OCUM-1	poorly differentiated adenocarcinoma containing signet ring cells;	pleural effusion	[6]
6	RERF-GC-1B	Adenocarcinoma;	pyloric lymph nodes	[7]
7	SNU16	gastric carcinoma; poorly differentiated	metastatic ascites	[8]
8	YCC-21	Adenocarcinoma	ascites	[9]
9	YCC-22	Adenocarcinoma	ascites	[9]
10	YCC-3	Poorly differentiated	ascites	[10]
11	YCC-7	Adenocarcinoma	ascites	[10]

Supplementary Table 3: Genes associated with top predicted super-enhancers (in green). Genes that were used in evaluating patient survival were indicated as "Yes".

Genes associated with top N super-enhancers	N=60	N=50	N=30
ABTB2	Yes	Yes	Yes
AC104654.2			
AGFG2			
AP000344.3			
ATP2C2			
ATP6V1C2			
BAIAP2L1	Yes	Yes	
BFSP2			
BX470102.3			
CAMK2N1	Yes	Yes	
CCAT1			
CCDC88C			
CDH17	Yes	Yes	
CDKN2B	Yes		
CLDN1	Yes	Yes	Yes
CLRN3	Yes	Yes	Yes
CREB3L1	Yes	Yes	Yes
DSG2	Yes	Yes	
EGFR	Yes	Yes	Yes
EPHB2	Yes	Yes	Yes
ETV4	Yes	Yes	Yes
GDA	Yes	Yes	Yes
GDPD5	Yes	Yes	
GLS	Yes	Yes	Yes
HRH1			
IGFL4			
IL22RA1			
ITPK1	Yes	Yes	Yes
KB-1471A8.1			
KIAA1211			
LAMC2	Yes		
LINC00299			
MALL	Yes		
MMP20			
MYO16-AS1			
NOTCH1	Yes		
PCSK5			
PDP1			
PFKP	Yes		
RARRES1	Yes	Yes	Yes
RBCK1	Yes	Yes	Yes
RNF170	Yes	Yes	
RP11-400N13.2			
RP11-486A14.1			
SLC9A4			
SMURF1	Yes	Yes	Yes
SOX13	Yes	Yes	
SSTR5			
ST3GAL4	Yes	Yes	
TASP1	Yes	Yes	Yes
TPCN2	Yes	Yes	Yes
TPRXL			Yes
TTYH3	Yes	Yes	
UPP1	Yes	Yes	
VIPR1	Yes	Yes	
ZKSCAN1			
ZNRF3			

Supplementary Table 4: Outer primer pairs (4C-seq) for selected regions of interest.

Region Coordinates	Forward Primer	Reverse Primer
chr18:20673650-20703450	GTCTGCGCCTCAGGAAAAT	AAGGCTGTTTCCTGTCTTGG
chr13:73,969,600-74,042,900	CCCTACCACTTTCCCTTTTC	TATGCAAGGGCATCAATTAGG
chr1:201,985,650-202,089,000	CTTGAGGAACACAGAAGGGC	CAGCACTCTGCAAACAGACT
chr6:137,279,250-137,292,400	AAAGTCTCTGCCATCTCCCAG	AAATCAAAGCTCAGAGGACTGG

Supplementary Table 5: Nested primer pairs for selected regions of interest. Red font indicates Nextera® Index Kit – PCR primers; blue font indicates i5 indices (N502); gold font indicates i7 indices (from top to bottom, N705 to N708); green font indicates Nextera® transposase sequences and black font indicates designed nested primer sequences.

Region Coordinates	Forward Primer	Reverse Primer
chr18:20673650 - 20703450	AATGATACGGCGACCACCGAGATCTACACCTCTCTATTCGTCGGCAGCGTCAGATGTGTATAAGAGACAGTTGTCCATCCCCATATCTTGG	CAAGCAGAAGACGGCATACGAGATAGGAGTCCGTCTCGTGGGCTCGGAGATGTGTATAAGAGACAGAGGCTGTTTCCTGTCTTGGG
chr13:73,969,600 - 74,042,900	AATGATACGGCGACCACCGAGATCTACACCTCTCTATTCGTCGGCAGCGTCAGATGTGTATAAGAGACAGGTCAACTGAATCAAGACATAAATTC	CAAGCAGAAGACGGCATACGAGATCATGCCTAGTCTCGTGGGCTCGGAGATGTGTATAAGAGACAGGGGAGTCCTTCAGTGTAACC
chr1:201,985,650 - 202,089,000	AATGATACGGCGACCACCGAGATCTACACCTCTCTATTCGTCGGCAGCGTCAGATGTGTATAAGAGACAGCCCCACTTCCCATTTCCCAA	CAAGCAGAAGACGGCATACGAGATGTAGAGAGGTCTCGTGGGCTCGGAGATGTGTATAAGAGACAGACCTAACAAAGCGCTCAGCT
chr6:137,279,250 - 137,292,400	AATGATACGGCGACCACCGAGATCTACACCTCTCTATTCGTCGGCAGCGTCAGATGTGTATAAGAGACAGGTTGCTTTGGCTAACTATTTGGA	CAAGCAGAAGACGGCATACGAGATCCTCTCTGGTCTCGTGGGCTCGGAGATGTGTATAAGAGACAGTAATAGCCTTGGTCCCCAGG

Supplementary Table 6: Primers used in enhancer deletion using CRISPR/Cas9 genome editing technology.

Purpose	Oligo name	Sequence
Construct CRISPR sgRNA plasmids	e1-5'-1	CACCgCCCCATGTCCCCATACAGGC
	e1-5'-2	AAACGCCTGTATGGGGACATGGGGc
	e1-3'-1	CACCGGCACACCAGGCAGGATTCC
	e1-3'-2	AAACGGAATCCTGCCTGGTGTGCC
	e2-5'-1	CACCgCGGGACTCAGACCTTAGTCA
	e2-5'-2	AAACTGACTAAGGTCTGAGTCCCGc
	e2-3'-1	CACCGAGGATTTCTTAAGCCCAGA
	e2-3'-2	AAACTCTGGGCTTAAGAAATCCTC
	e3-5'-1	CACCgTGAGGGAGGATAGGCGGGCC
	e3-5'-2	AAACGGCCCGCCTATCCTCCCTCAc
	e3-3'-1	CACCgCACCTAGAGGCCTGCTTTAG
	e3-3'-2	AAACCTAAAGCAGGCCTCTAGGTGc
	e4-5'-1	CACCGAAGAGAACTCCACCGGGTG
	e4-5'-2	AAACCACCCGGTGGAGTTCTCTTC
	e4-3'-1	CACCgCAGACATGACCTAGGTTCCC
	e4-3'-2	AAACGGGAACCTAGGTCATGTCTGc
Determine deletion of enhancer (PCR)	e1-5'-F	GCCTTCCCTTCCTGATGTC
	e1-3'-R	TAATGGCAAGACTGGTATCCAC
	e2-5'-F	CTTGTGGTACTGTTCCCAGAC
	e2-3'-R	CAGCCTGGGAAGCATATTGA
	e3-5'-F	CTGGGTTCCCACCTGATAAT
	e3-3'-R	GATGAAATCCAAGTCATTGTGTCC
	e4-5'-F	CTTCTGGGTTCAAGTGAGTCT
	e4-3'-R	CATGAGCAAAGGTCCTCCTAC
Determine retention of enhancer after targeting (qPCR)	e1-int-F	CAGTAGGTACACCTGGCAATAG
	e1-int-R	ATCCTGCTTCCTCTTGGAATATC
	e2-int-F	CCAGCTTCTTTCCTCTCCTTATC
	e2-int-R	GGTGAAATCCCATCTCCACTAAA
	e3-int-F	ATCCAGACACACCTGTAGGA
	e3-int-R	CAGAACAAAGTCCAGAGAGAGG
	e4-int-F	CCTGCCTCTCTTCTGCTTTC
	e4-int-R	GTTCATGCCCTGCCTTATCT

Supplementary Discussion

Correlation between gene expression and distal predicted **regulatory elements**

To correlate distal predicted regulatory elements defined by Nano-ChIPseq to gene expression, we identified 80 predicted super-enhancers exhibiting high recurrence across multiple lines (P < 0.0001, empirical test). The same approach was also used to identify highly recurrent predicted typical enhancers. For both predicted super-enhancers and predicted typical enhancers, genes associated with distal regulatory elements exhibited higher expression that randomly selected genes (Supplementary Fig. 4b). Comparing the expression of predicted super-enhancer/typical enhancer associated genes revealed higher overall expression levels (in unit of percentile) for predicted super-enhancer associated genes ($P = 5.2x10^{-3}$, one-sided Wilcoxon's rank sum test). These results suggest a positive association between H3K27ac enrichment in predicted super-enhancers and predicted typical enhancers with target gene expression.

Comparisons of primary gastric non-malignant samples to Epigenome Roadmap

To confirm that our non-malignant gastric tissues are indeed reflective of gastric epithelia and not muscle, immune cells etc, we compared the non-malignant gastric H3K27ac profiles from our study to previously published normal gastric profiles and also to stomach smooth muscle profiles[11]. For each Nano-ChIPseq profile, 70% (average) of the H3K27ac signals overlapped with published normal gastric profiles, while only 34% (average) overlapped with stomach smooth muscle. The result suggests that our non-malignant gastric samples are indeed reflective of gastric epithelia and not stomach smooth muscle.

Associations between copy number alterations and predicted **super-enhancers in gastric cancer**

We investigated the extent to which recurrent somatic altered predicted super-enhancers might be associated with somatic copy number alterations (sCNAs). We computed overlaps between the predicted super-enhancers and copy number information from the cell lines and primary GCs, using in-house generated Affymetrix SNP6.0 array data. Our analysis was restricted to regions covered by at least 6 SNP probes per 10 kb (2x higher than the mean genome-wide coverage), to allow regions of sCNA to be confidently identified. Confirming the reliability of our sCNA analysis, an average of 98% of copy number gains and 82% of copy number losses in our analysis were also reported in Cancer Cell Line Encyclopedia[12] for GC cell lines found in the latter (FU97, KATO-III, MKN7, OCUM-1, RERFGC1B, SNU16).

In the cell lines, we found that only 5~6% (± 6% standard deviation) of the predicted super-enhancers were associated with copy number gains (average log2 ratio > 0.6). For example, an *FGFR2*-associated predicted super-enhancer detected in KATO-III overlapped with copy number gain (Supplementary Fig. 8b), suggesting that the observed higher H3K27ac read density at the locus is potentially driven by regional genomic amplification. On the other hand, the majority of the predicted super-enhancers detected in GC cell lines localized at copy number neutral regions, suggesting that the establishment of predicted super-enhancers is independent of somatic copy number events. This fraction is greater than by random chance (P< 0.01, empirical test)

Similarly, in primary GCs, we were able to compute CNA/SE correlations for 1,748 recurrent somatic gain predicted super-enhancers in 19 primary T/N pairs. We found that a only small fraction of somatic gain predicted super-enhancers (< 2% ± 3% s.d) overlapped with copy number gains (Supplementary Fig. 8c), with >90% of somatic gain predicted super-enhancers found in individual T/N pairs are detected within copy number neutral regions (Supplementary Fig. 8a). This result suggests that there is no strong association between somatic gain of H3K27ac in predicted super-enhancers and copy number changes[13], and that H3K27ac acquisition at predicted super-enhancers in the tumor samples are likely driven by mechanisms separate from copy number alteration.

Supplementary References

1. Baek, S.J. *et al.* Integrated epigenomic analyses of enhancer as well as promoter regions in gastric cancer. *Oncotarget* (2016).
2. Terashima, M. *et al.* Establishment of an alpha-fetoprotein-producing gastric cancer cell line in serum-free media. *Jpn J Cancer Res* **82**, 883-5 (1991).
3. Sekiguchi, M., Sakakibara, K. & Fujii, G. Establishment of cultured cell lines derived from a human gastric carcinoma. *Jpn J Exp Med* **48**, 61-8 (1978).
4. Yokozaki, H. Molecular characteristics of eight gastric cancer cell lines established in Japan. *Pathol Int* **50**, 767-77 (2000).
5. Ku, J.L. *et al.* Establishment and characterization of six human gastric carcinoma cell lines, including one naturally infected with Epstein-Barr virus. *Cell Oncol (Dordr)* **35**, 127-36 (2012).
6. Kubo, T. [Establishment and characterization of a new gastric cancer cell line (OCUM-1), derived from Borrmann type IV tumor]. *Nihon Geka Gakkai Zasshi* **92**, 1451-60 (1991).
7. Klijn, C. *et al.* A comprehensive transcriptional portrait of human cancer cell lines. *Nat Biotechnol* **33**, 306-12 (2015).
8. Park, J.G. *et al.* Characteristics of cell lines established from human gastric carcinoma. *Cancer Res* **50**, 2773-80 (1990).
9. Ooi, C.H. *et al.* Oncogenic pathway combinations predict clinical prognosis in gastric cancer. *PLoS Genet* **5**, e1000676 (2009).
10. Rha, S.Y. *et al.* Modulation of biological phenotypes for tumor growth and metastasis by target-specific biological inhibitors in gastric cancer. *Int J Mol Med* **4**, 203-12 (1999).

11. Roadmap Epigenomics, C. *et al.* Integrative analysis of 111 reference human epigenomes. *Nature* **518**, 317-30 (2015).
12. Barretina, J. *et al.* The Cancer Cell Line Encyclopedia enables predictive modelling of anticancer drug sensitivity. *Nature* **483**, 603-7 (2012).
13. Akhtar-Zaidi, B. *et al.* Epigenomic enhancer profiling defines a signature of colon cancer. *Science* **336**, 736-9 (2012).
